# Independent and Combined Effects of Obesity and Cardiovascular Diseases on the Risk of Cognitive Impairment and Dementia: A Systematic Review and Meta-Analysis of Prospective Cohort Studies Involving 8,276,914 Participants

**DOI:** 10.3390/ijms27041892

**Published:** 2026-02-16

**Authors:** Getu Gamo Sagaro, Seyed Khosrow Tayebati

**Affiliations:** 1 School of Pharmaceutical and Health Products Sciences, University of Camerino, 62032 Camerino, Italy; 2 School of Public Health, College of Health Sciences and Medicine, Wolaita Sodo University, Sodo 138, Ethiopia

**Keywords:** dementia, cognitive impairment, obesity, central obesity, abdominal obesity, body mass index, cognitive function, waist circumference

## Abstract

Background: Dementia and cognitive impairment are increasing worldwide, particularly in older adults, imposing substantial health and societal burdens. Obesity and cardiovascular diseases (CVDs) are recognized risk factors; however, existing evidence is inconsistent, and their combined effects remain poorly understood. This study aimed to assess the independent and joint associations of obesity and CVDs with the risk of cognitive impairment and dementia through a systematic review and meta-analysis. Methods: A comprehensive literature search was conducted across three major electronic databases (PubMed, Web of Science, and Scopus) to identify relevant studies published from January 2015 through 30 June, 2025. A random-effects meta-analysis was performed to estimate the independent effects of obesity and CVDs on the outcome of interest, reporting the pooled hazard ratios (HRs) and 95% confidence intervals (CIs). The risk of bias was assessed using the Newcastle–Ottawa Scale (NOS), and the certainty of the evidence was evaluated using the GRADE approach. Results: A total of 25 studies comprising 8,276,914 participants were included. Body mass index (BMI)-defined obesity was associated with a lower risk of cognitive impairment (pooled HR = 0.85; 95% CI: 0.74–0.98; I^2^ = 40.5%) but showed no association with incident dementia (pooled HR = 1.00; 95% CI: 0.86–1.15; I^2^ = 96%). In contrast, central obesity, assessed by waist circumference (WC), was associated with a 14% increased risk of dementia (pooled HR = 1.14; 95% CI: 1.03–1.27; I^2^ = 96%). Coronary heart disease, stroke, and atrial fibrillation were each significantly associated with dementia risk, with pooled HRs of 1.41 (95% CI: 1.29 –1.54; I^2^ = 0%), 1.53 (95% CI: 1.35–1.74; I^2^ = 37%), and 1.30 (95% CI: 1.12–1.50; I^2^ = 68.8%), respectively. Evidence on the joint effects of obesity and CVD was limited to a single study, which reported that men with both conditions had a 58% higher risk of all-cause dementia compared with those of normal weight without CVD (HR = 1.58; 95% CI: 1.37–1.81). Conclusion: BMI-defined obesity was inversely associated with cognitive impairment, whereas central obesity was associated with an increased risk of dementia. Among CVDs, stroke showed the strongest association with incident dementia. However, the overall certainty of evidence across these findings was low, and these findings should be interpreted with caution.

## 1. Introduction

Cognitive impairment (CI) refers to a condition characterized by a decline in cognitive abilities that affects an individual’s ability to think, reason, make decisions, and carry out daily activities [1]. Cognitive impairment is not just an age-related disorder; it is a major health and social concern that affects individuals, families, and communities [2]. CI can lead to a significant loss of memory and learning abilities, increasing dependency and social isolation [3]. It ranges from mild to severe, with varying effects on an individual’s daily functioning and quality of life [1]. It is prevalent among the elderly population and increases with age [4,5]. Cognitive impairment exists along a spectrum of decline, with mild cognitive impairment (MCI) serving as the transitional stage between normal age-related changes and dementia. When MCI advances, it may develop into dementia, leading to profound cognitive decline and diminished independence. A study reported that people with MCI face a notably higher likelihood of progressing to dementia, with reported conversion rates reaching up to 15% [6]. Recent studies indicate that the magnitude and severity of cognitive loss have been rising in recent years [7]. In 2015, the global prevalence of dementia was estimated at 46.8 million individuals. This figure is projected to increase to 74.7 million by 2030 and 131.5 million by 2050 [8].

According to the World Health Organization (WHO), more than 55 million people worldwide live with CI, and nearly 10 million new cases are diagnosed each year [9]. Research indicates that CI is not driven by a single cause but rather a multifactorial condition influenced by diverse and interacting factors. Studies have shown that genetics, environmental exposures, lifestyle choices, and underlying health conditions all play important roles in its onset and progression [10,11]. Additionally, biochemical imbalances (such as low vitamin B12, low folate, and high homocysteine) and physiological conditions, including dyslipidemia, hypertension, diabetes, and obesity, significantly increase the risk of cognitive impairment [10,11,12,13]. The link between CVDs, especially atrial fibrillation (AF), coronary heart disease (CHD), heart failure (HF), and stroke, and cognitive impairment (CI) has been well documented [14,15,16,17,18,19]; the association is even more strongly supported when considering their role in increasing the risk of dementia. The evidence on whether CVDs independently increase the risk of cognitive impairment (CI) remains inconsistent. While some studies have identified CHD as a risk factor for CI [20,21,22], more recent investigations have found no significant association between the two conditions [23,24]. Previous studies have reported a positive association between obesity and the risk of developing midlife dementia [25,26]. Other research has suggested that obesity, measured through body mass index (BMI), waist circumference (WC), and waist-to-hip ratio (WHR), is linked to an increased risk of cognitive impairment or dementia [26,27]. However, some studies have found no significant association between increased adiposity or central obesity and the incidence of dementia [28,29]. These conflicting findings indicate that the relationship between obesity and cognitive decline remains unclear. Moreover, the combined effects of obesity and CVD on the risk of cognitive impairment and incident dementia remain poorly understood. Furthermore, evidence on their synergistic impact on cognitive outcomes is still limited. Previous studies have shown that cardiometabolic multimorbidity (CMD), defined as the coexistence of two or more conditions, such as type 2 diabetes, heart disease, stroke, hypertension, or coronary artery disease (CAD), is linked to accelerated cognitive decline and an increased risk of both cognitive impairment and dementia [30,31,32,33,34,35]. However, obesity is typically excluded from this definition. Thus, examining the combined contribution of obesity and CVDs is crucial for improving our understanding of the metabolic and vascular pathways underlying cognitive aging and dementia risk.

The present study aimed to examine the independent and combined effects of obesity and CVD on cognitive impairment and incident dementia. We hypothesized that obesity and CVD independently increase the risk of cognitive impairment and incident dementia among adults aged 40 years and older at baseline. Furthermore, individuals with both conditions were expected to have a higher risk than those with either condition alone or neither. We also hypothesized that these associations vary by age at baseline, with midlife obesity (ages 40–59) showing a stronger association with subsequent dementia than obesity in later life (age ≥ 60). Understanding the independent and joint contributions of obesity and CVD to cognitive impairment is essential for identifying high-risk individuals and for developing effective prevention and intervention strategies that address both metabolic and cardiovascular health. These findings may also inform public health policies and clinical practices aimed at reducing the global burden of cognitive impairment by targeting multiple modifiable risk factors simultaneously.

## 2. Materials and Methods

This systematic review was performed following the Preferred Items for Systematic Reviews and Meta-Analyses (PRISMA) reporting standards [36]. A protocol for this study has been registered with the International Prospective Register of Systematic Reviews (PROSPERO) (CRD420251060451).

### 2.1. Search Strategy and Data Sources

We conducted a comprehensive search in the PubMed, Scopus, and Web of Science databases for relevant peer-reviewed studies published from 1 January 2015 to 30 June 2025. The key search terms used in this study were as follows: (1) Obesity-related terms, including body mass index (BMI), waist circumference (WC), waist-to-hip ratio (WHR), central obesity, and abdominal obesity; (2) Cardiovascular-related terms, including coronary heart disease (CHD), heart failure (HF), coronary artery disease (CAD), myocardial infarction (MI), atrial fibrillation (AF), stroke/cerebrovascular diseases, and CVD; (3) Cognitive-related terms, including cognitive impairment (CI), cognitive deficits, cognitive decline, cognitive function, cognition, dementia, and Alzheimer’s disease (AD).

Key terms were combined using Boolean operators such as “AND” and “OR,” and the search results were limited to studies involving humans. To account for different word forms, group related terms, and effectively link concepts, we used advanced search techniques. These included the use of quotation marks, parentheses, and truncation symbols alongside key terms. We searched for studies evaluating the association between obesity and incident cognitive impairment or dementia using the search terms obesity AND cognition (1 AND 3). For the second objective, to identify studies assessing the link between CVD and cognitive outcomes, we used the terms cardiovascular AND cognition (2 AND 3). To find studies investigating the combined effects of obesity and CVD on cognitive impairment or dementia, we used the search terms obesity AND cardiovascular AND cognition (1 AND 2 AND 3). The complete list of search terms used for each objective across the selected databases is provided in the Supplementary Materials (Table S1).

### 2.2. Eligibility Criteria and Selection of Studies

Studies were considered if they met the following criteria: (1) Original prospective cohort or longitudinal studies with a follow-up period of more than one year, investigating the association between baseline obesity and CVDs, either independently or synergistically with the incidence of cognitive impairment and dementia. (2) For obesity-related cognitive impairment or incident dementia, studies that evaluated baseline obesity using categorical measures such as BMI, WC, or WHR based on WHO guidelines or other recognized standards were included. For BMI, obesity was compared to a normal-weight reference group, whereas for WC and WHR, the lowest category served as the reference. (3) Studies evaluating the association between CVDs and cognitive impairment, using participants without CVDs as the comparison group. (4) Studies examining synergistic effects (obesity and CVD) comparison groups included individuals with neither obesity nor CVD, or those with only obesity or only CVD. (5) Studies reported hazard ratios (HR), relative risks (RR), or odds ratios (OR), or data to compute them for obesity, CVDs, and combined exposure. (6) Studies published in a peer-reviewed journal and written in English. (7) Studies that included individuals aged 40 years or older at baseline. (8) Studies that clearly defined baseline CVDs, including CHD, HF, stroke, AF, or myocardial infarction (MI), using standardized methods such as WHO criteria, ICD-9/10 codes, electrocardiography (ECG), clinical examinations, national patient registries (NPR), or medical records. (9) Studies that clearly diagnosed cognitive impairment or dementia (the primary outcomes of this study) using neurological or cognitive assessments or established diagnostic criteria such as the Diagnostic and Statistical Manual of Mental Disorders (DSM), the National Institute of Neurological and Communicative Disorders and Stroke–Alzheimer’s Disease and Related Disorders Association (NINCDS-ADRDA) criteria, ICD-9 or ICD-10 codes, the Alzheimer’s Disease Assessment Scale–Cognitive Subscale (ADAS-Cog), or the Mini-Mental State Examination (MMSE). On the other hand, studies were excluded if they: (1) employed a retrospective cohort, cross-sectional, case–control, or clinical design, or involved longitudinal trajectories; (2) were randomized controlled trials and studies derived from clinical trial cohorts, even if the exposure was analyzed observationally; (3) were reviews, including systematic reviews, meta-analyses, or other review types; (4) were case studies, conference abstracts, unpublished documents, letters to the editor, or book chapters; (5) lacked clearly defined outcomes; (6) were duplicate or retracted publications; (7) used continuous obesity measures such as BMI, WC, or WHR; (8) were studies that updated exposure status during follow-up (time-dependent exposures); or (9) did not meet our predefined eligibility criteria. The PICOS eligibility criteria are provided in the Supplementary Materials (Table S2).

First, all retrieved records were imported into a Zotero library (Center for History and New Media, George Mason University) for organization and deduplication. Two reviewers (GGS and SKT) independently screened titles, abstracts, and full texts for relevance according to predefined eligibility criteria. Any discrepancies were resolved through discussion.

### 2.3. Data Extraction

One author (GGS) extracted data from the included studies, and a second author (SKT) cross-checked all retrieved data. Using a standard data extraction sheet, we collected the following study characteristics for each included study: author name, publication year, country of origin, study design, follow-up duration, characteristics of the study population (mean age, sex, and sample size), baseline participant characteristics (obesity measurement, types of CVDs, and reference group), ascertainment procedures for exposure variables (obesity and CVD), number of incident outcomes (cognitive impairment or dementia), diagnostic criteria for outcomes, confounders adjusted for, and main results (hazard ratio/relative risk values) of the most adjusted models for meta-analysis.

### 2.4. Assessment of Risk of Bias

Two investigators (GGS and SKT) independently evaluated the methodological quality of the selected studies using the Newcastle–Ottawa Scale (NOS) [37]. According to the NOC scale, each study was rated on nine items, with a total of nine possible points distributed across selection (up to 4 stars), comparability (up to 2 stars), and outcome (up to 3 stars). Using the predefined scoring range of 0–9, we classified the included studies as low (0–4), moderate (5–6), or high quality (7–9). Disagreements were resolved through discussion between the authors.

### 2.5. Assessment of the Certainty of Evidence

We assessed the certainty of the evidence using the Grading of Recommendations, Assessment, Development and Evaluation (GRADE) approach [38] and developed Summary of Findings tables with GRADEpro GDT (McMaster University and Evidence Prime Inc.). GRADE offers a comprehensive framework for assessing the quality and reliability of scientific evidence across domains such as risk of bias, inconsistency, indirectness, imprecision, publication bias, and others. According to the GRADE framework, each outcome is classified into four distinct levels of evidence certainty: high, moderate, low, and very low, which reflect the degree of confidence in the accuracy of the estimated effects. Evidence derived from cohort studies begins with a grade of “Low.” Grading helps determine the robustness of the findings and their potential relevance to clinical and public health settings.

### 2.6. Statistical Analysis

The data analysis was conducted using the *R programming language* (*Version 4.3.0*) [39]. The meta-analysis was performed using the *metagen()* function from the *meta* package [40]. We utilized multivariable-adjusted HRs or relative risks (RRs) as provided in the original studies. Fixed-effect or random-effects models were used, as appropriate, to estimate pooled effect sizes (HR or RR) and their corresponding 95% confidence intervals (CIs) for the incidence of cognitive impairment or dementia. In the case of the fixed-effect model, the pooled HR was calculated by averaging log (HR) or log (RR) weighted by the inverse of their variances. In contrast, the random-effect model accounts for potential between-study heterogeneity. DerSimonian and Laird’s method was used to estimate the between-study variance (τ^2^), which is incorporated into the weights used to calculate the pooled log (HR) or log (RR) [41]. We reported pooled risk estimates using a random-effects model if heterogeneity was significant, indicated by a Q-test *p*-value < 0.10 [42].

We combined the two sex-specific adjusted effects of obesity-versus-normal BMI within each study into a single study-level effect when studies reported sex-segmented adjusted obesity HR or RR (individual estimates for males and females versus the same normal-BMI reference). Before pooling, sex-specific HRs or RRs were log-transformed, and their standard errors were calculated from reported 95% confidence intervals. We then computed an inverse-variance fixed-effects weighted mean of the log-HRs, assuming independence between the male and female strata, so that no within-study covariance was needed [42]. On the other hand, when studies reported multiple adjusted HRs or RRs for obesity defined by BMI or WC, for example, BMI 30–34.9 and ≥35 kg/m^2^, each versus the same normal-weight reference, or several WC categories compared with the same lowest group, we combined these within-study estimates into single *obesity-versus-normal BMI* and *high-versus-low WC* contrasts before performing the meta-analysis. Adjusted HRs/RRs were log-transformed, and standard errors were derived from 95% confidence intervals. When multiple contrasts shared a common reference, we used an inverse-variance fixed-effect model that accounted for covariance between categories. When category-specific counts or person-time data were available, we combined categories by summing events and denominators to reconstruct a single, comparable effect estimate [43,44,45]. Moreover, in cases of multiple reports from a single cohort, we selected the one with the longest follow-up period [46,47].

We assessed the heterogeneity in the meta-analysis using the p-value from the chi-square (χ^2^) test, considering heterogeneity statistically significant if *p* < 0.10 [42]. The magnitude of heterogeneity was quantified using the I^2^ statistic, which represents the percentage of variability in effect estimates that is due to heterogeneity rather than chance [42]. The degree of heterogeneity was interpreted according to I^2^ values as follows: 0–40% (might not be important), 30–60% (may represent moderate heterogeneity), 50–90% (may represent substantial heterogeneity), and 75–100% (considerable heterogeneity), with overlapping ranges as suggested by Cochrane [42].

We further conducted subgroup analyses to explore whether heterogeneity in hazard ratios could be attributed to differences in obesity definitions, age categories, sex, follow-up duration, or publication year. Sensitivity analyses were also undertaken to assess the robustness of the pooled estimates by sequentially removing the most influential study and recalculating the hazard ratios for the remaining studies. Egger’s regression test [48] and visual inspection of the funnel plot [49] were performed when appropriate to assess potential publication bias.

## 3. Results

### 3.1. Literature Search

**Figure 1:**
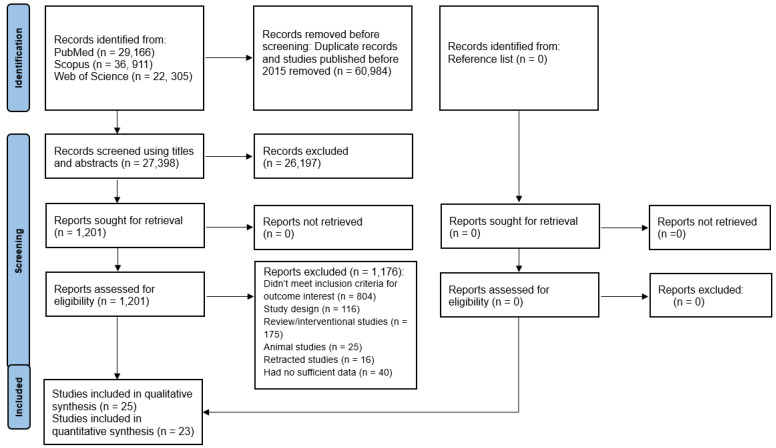
PRISMA flow diagram.

Across the three search engines, 88,382 records were initially retrieved. After removal of duplicates and studies published before 2015, 27,398 records remained for screening. Following title and abstract screening, 26,197 records were excluded because they were reviews (systematic, meta-analyses, narrative, or scoping reviews), conference abstracts, book chapters, letters, editorials, had inappropriate study designs, or were unrelated to the research question. A total of 1201 full-text articles were assessed for eligibility, of which 1176 were excluded for reasons including animal studies, review articles, interventional designs, retracted publications, inadequate study design, or insufficient data. Ultimately, 25 prospective cohort studies were included in the qualitative synthesis [26,31,32,50,51,52,53,54,55,56,57,58,59,60,61,62,63,64,65,66,67,68,69,70,71], and 23 of these were included in the quantitative synthesis (meta-analysis). The study selection process is illustrated in the PRISMA flow diagram (Figure 1).

### 3.2. Characteristics of Included Studies

In the qualitative synthesis, twenty-five studies were included, involving a total of 8,276,914 participants with a mean age at baseline ranging from 49.3 to 81.23 years. Sixteen studies, encompassing 7,040,489 participants, provided data on the relationship between obesity measured by BMI and WC and the risk of cognitive impairment and dementia [26,50,51,52,53,54,55,56,57,58,59,60,61,62,63,64]. Eight studies with 771,809 participants examined the association between CVDs and the risk of cognitive impairment or dementia [31,32,65,67,68,69,70,71]. Additionally, one study with 464,616 participants evaluated the combined effects of BMI-defined obesity and any CVDs on dementia risk [66]. The studies were conducted between 2015 and 2025, with the majority carried out geographically in Europe (46.2%), followed by Asia (30.8%) and the Americas (23%). Sample sizes ranged from 1519 to 4,106,590, and average follow-up periods varied from 3.8 to 23 years. During follow-up, 269,812 participants who were cognitively normal at baseline were subsequently diagnosed with dementia; 10,649 with cognitive impairment; 7353 with Alzheimer’s disease; and 2738 with vascular dementia. The detailed characteristics of the included studies are presented in Table 1.

### 3.3. Quantitative Synthesis

#### 3.3.1. Association Between BMI-Defined Obesity and Risk of Cognitive Impairment and Dementia

**Figure 2:**
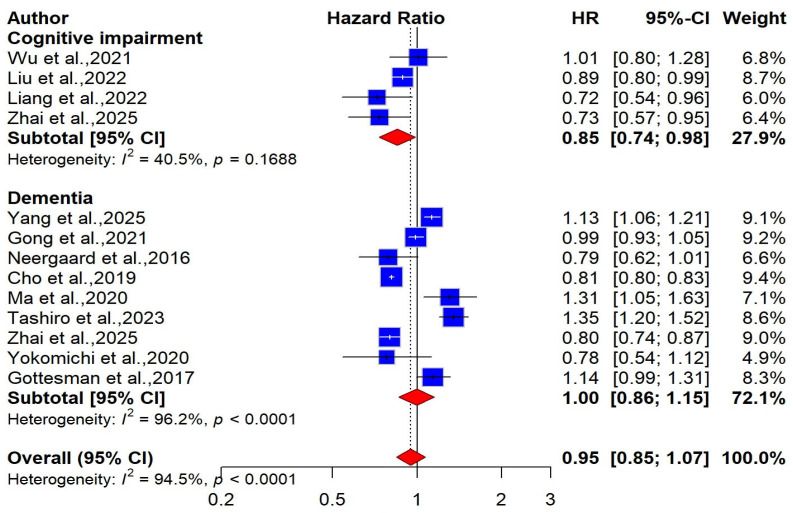
Forest plot of the association between BMI-defined obesity and risk of cognitive impairment and dementia [26,50,51,52,53,54,55,56,57,58,59,60].

Regarding the risk of cognitive impairment, four studies involving a total of 50,872 participants provided data on the association between BMI-defined obesity and cognitive impairment [50,51,52,58]. The pooled hazard ratio (HR) for this association was 0.85 (95% CI: 0.74–0.98; I^2^ = 40.5%), indicating a statistically significant inverse association (Figure 2). This corresponds to an approximately 15% lower risk of cognitive impairment among individuals with obesity compared with those with normal BMI. However, the certainty of the evidence was rated as low, suggesting that the observed association should be interpreted with caution, as it may be influenced by methodological limitations or residual confounding.

**Figure 3:**
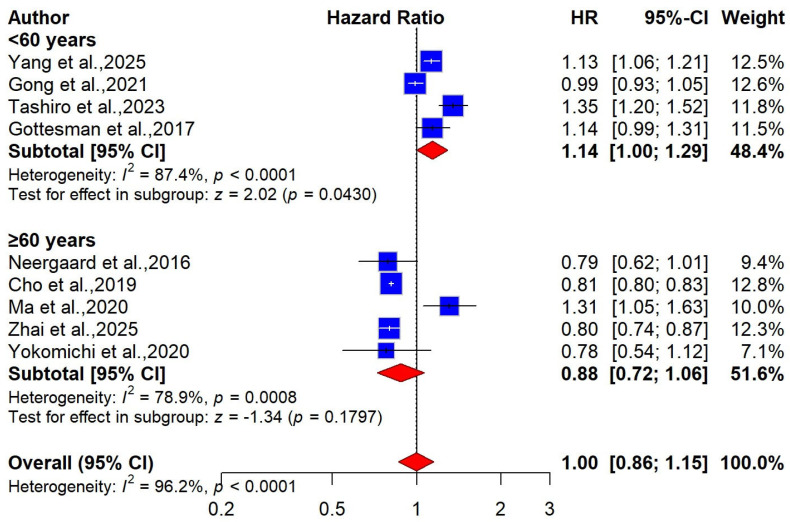
Forest plot of the association between BMI-defined obesity and risk of dementia, stratified by age group [26,53,54,55,56,57,58,59,60].

Nine studies involving 2,430,710 participants were included in a meta-analysis examining the relationship between obesity and the risk of dementia [26,53,54,55,56,57,58,59,60]. Our combined analysis showed no significant relationship between BMI-defined obesity and dementia risk using a random-effects model [pooled HR = 1.00 (95% CI: 0.86 to 1.15), I^2^ = 96.2%, very low certainty, Figure 2]. We performed a subgroup analysis to better understand how BMI-obesity affects dementia risk and to identify potential sources of heterogeneity. Accordingly, nine studies were divided into two groups based on the participants’ age: midlife (<60 years) and late life (≥60 years). Of these studies, 4 with 1,519,583 participants assessed midlife BMI-defined obesity [53,54,57,60], while 5 studies with 911,127 participants evaluated late-life BMI-defined obesity [26,55,56,58,59]. In our meta-analysis, we found that midlife obesity may be associated with a slightly increased risk of late dementia [pooled HR = 1.14 (95% CI: 1.00 to 1.29), I^2^ = 87.4%]. On the other hand, no significant association was observed in late life [pooled HR = 0.88 (95% CI: 0.72 to 1.06), I^2^ = 78.9%, Figure 3]. For dementia subtypes, three studies involving 1,472,274 participants were included in the meta-analysis. The pooled findings showed no significant association between BMI-defined obesity and Alzheimer’s disease (AD) risk [pooled HR = 0.85, 95% CI: 0.65—1.12; I^2^ = 94.3%, very low certainty], or with vascular dementia risk [pooled HR = 1.15, 95% CI: 0.90–1.45; I^2^ = 83.9%, very low certainty] (Supplementary Figure S1A). We conducted sex-stratified pooled analyses, which showed no significant association between BMI-defined obesity and dementia risk in either women [pooled HR = 1.02, 95% CI: 0.87–1.20; I^2^ = 96.8%] or men [pooled HR = 0.97, 95% CI: 0.80–1.17; I^2^ = 90.2%] (Supplementary Figure S1B).

We also carried out subgroup analyses stratified by mean follow-up duration (<10 years vs. ≥10 years) and by the BMI cut-off values used to define obesity (lower cut-off ≥25 kg/m^2^ vs. higher cut-off ≥30 kg/m^2^). Across all subgroups, the pooled estimates showed no significant associations, regardless of the BMI cut-off applied or the length of follow-up. The corresponding hazard ratios and additional analytic results are provided in Supplementary Figure S2 (Figure S2A,B). Our leave-one-out sensitivity analysis demonstrated that the combined estimates for the relationship between BMI-defined obesity and risk of dementia were not influenced by any single study (see Supplementary Table S3).

#### 3.3.2. Association Between Central Obesity and Risk of Cognitive Impairment and Dementia

**Figure 4:**
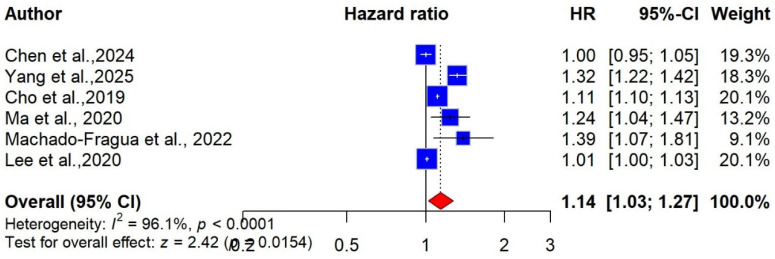
Forest plot of the association between WC-defined obesity and risk of dementia [26,53,56,61,63,64].

Seven studies involving 6,425,362 participants examined the association between central obesity, measured by WC, and the risk of cognitive impairment and dementia [26,53,56,61,62,63,64]. Of these, six studies (6,423,843 participants) specifically reported dementia outcomes and were eligible for the meta-analysis assessing the association between WC-defined obesity and dementia risk [26,53,56,61,63,64]. The pooled analysis showed that central obesity, measured by WC, was associated with a 14% increased risk of dementia compared with lower WC (pooled HR = 1.14 (95% CI: 1.03 to 1.27, I^2^ = 96%), with low certainty of the evidence (Figure 4).

**Figure 5:**
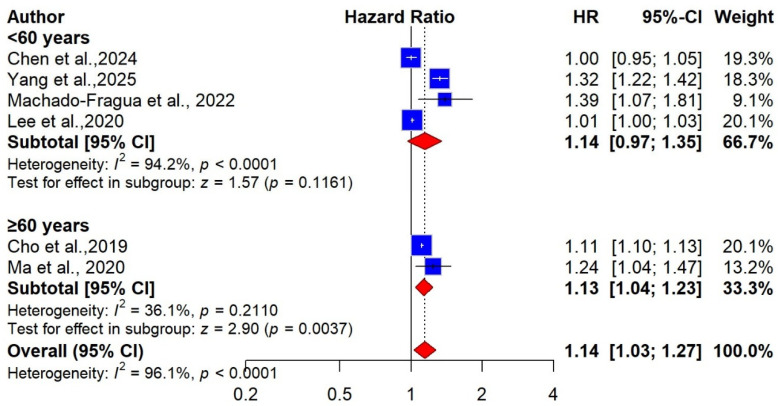
Forest plot of the association between central obesity and risk of dementia, stratified by age group [26,53,56,61,63,64].

We conducted several subgroup analyses to examine how central obesity may influence the risk of dementia. In terms of age at adiposity assessment, four studies with 5,545,179 participants contributed midlife WC data, and two studies with 878,664 participants contributed late-life WC data. According to our meta-analysis, for midlife, WC-defined obesity was not significantly associated with risk of dementia [pooled HR = 1.14 (95% CI: 0.97 to 1.35), I^2^ = 94%, Figure 5]. In contrast, in late life, it was associated with a 13% increased risk of dementia compared with low WC [pooled HR = 1.13 (95% CI: 1.04 to 1,23), I^2^ = 36%)(Figure 5]. Three studies provided sex-specific data [26,53,56]. Among women, three studies with 1,843,200 participants were included in the meta-analysis, and the pooled estimate indicated that WC-defined obesity was associated with a 24% increased risk of dementia compared with low WC (pooled HR = 1.24 (95% CI: 1.08 to 1.43), I^2^ = 92.5%). In comparison, for men, two studies with a total of 878,664 participants were available, and the combined results showed no significant association between central obesity and the risk of dementia (pooled HR = 1.03 (95% CI: 0.79 to 1.35), I^2^ = 61.4%) (see Supplementary Figure S3A). Furthermore, we conducted subgroup analyses based on mean follow-up duration (<10 years vs. ≥10 years). The pooled estimates indicated that WC-defined obesity was not significantly associated with dementia risk in participants followed for less than 10 years (pooled HR = 1.14; 95% CI: 0.98–1.32; I^2^ = 98.3%) or in those with follow-up periods of 10 years or longer (pooled HR = 1.16; 95% CI: 0.96–1.40; I^2^ = 81.4%), as shown in Supplementary Figure S3B.

**Figure 6:**
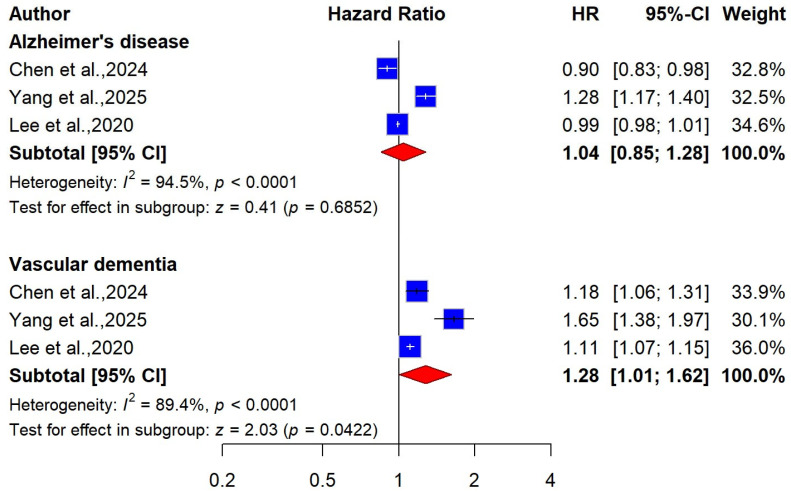
Forest plot of the association between WC-defined obesity and risk of dementia, stratified by dementia types [53,61,64].

For dementia subtypes, three studies encompassing 5,537,914 participants were included in the meta-analysis [53,61,64]. The pooled estimates showed no significant association between WC-defined obesity and Alzheimer’s disease (AD) (pooled HR = 1.04 (95% CI: 0.85 to 1.28, I^2^ = 94.5%)), with very low certainty of the evidence (Figure 6). Conversely, central obesity, measured by WC, was associated with a higher risk of vascular dementia (VaD) (HR = 1.28 (9% CI: 1.01 to 1.62, I^2^ = 89%), with low certainty of the evidence (Figure 6). Regarding cognitive impairment, a single study involving 1519 participants reported that higher WC was associated with a 41% increased risk (HR = 1.41 (95% CI: 1.01 to 1.98) [62].

#### 3.3.3. Association Between CVDs and Risk of Cognitive Impairment and Dementia

##### Coronary Heart Disease

**Figure 7:**
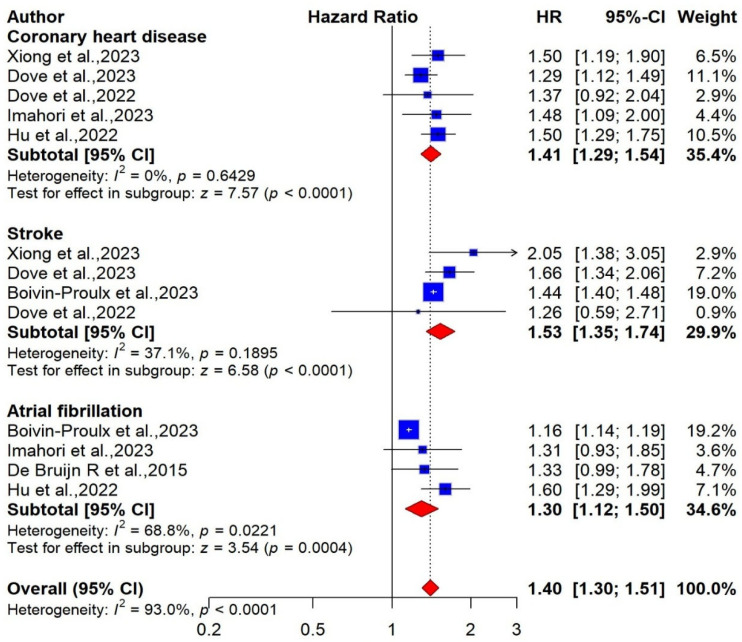
Forest plot of the association between CVDs and risk of dementia [31,32,65,67,69,70,71].

Five studies, including 439,375 participants, were included in the meta-analysis to investigate the association between CHD and the risk of dementia [31,32,65,70,71]. The pooled analysis showed that CHD was associated with an increased risk of dementia, using a random-effects model (pooled HR = 1.41; 95% CI: 1.29 to 1.54; I^2^ = 0%, low certainty) (Figure 7).

Two studies (N = 263,396) provided data on the association between CHD and dementia subtypes, including Alzheimer’s disease (AD) and vascular dementia (VaD) [31,71]. The meta-analysis indicated that CHD was associated with both VaD (pooled HR = 2.06; 95%CI: 1.03–4.13; I^2^ = 91%, Low certainty) and AD (pooled HR = 1.37; 95% CI: 1.15–1.62; I^2^ = 0%, Low certainty) (Supplementary Figure S4A). Due to insufficient data, subgroup analyses according to sex, mean follow-up duration, and age category were not performed.

##### Stroke

Four studies, involving a total of 511,954 participants, were included in the analysis of the association between stroke and dementia [31,32,65,67]. The pooled estimate showed that individuals with a history of stroke had a 53% higher risk of developing dementia compared with those with no history of stroke (pooled HR = 1.53; 95% CI: 1.35–1.74). Heterogeneity across studies was moderate (I^2^ = 37%), and the certainty of the evidence was rated as low (Figure 7). Additionally, two studies involving a total of 7163 participants examined the relationship between stroke and cognitive impairment [32,68]. The combined results indicated no statistically significant association between stroke and the risk of cognitive impairment (pooled HR = 1.06; 95% CI: 0.68–1.66; I^2^ = 55.6%), with the certainty of the evidence rated as low certainty (Supplementary Figure S4B).

##### Atrial Fibrillation

Four studies containing 575,195 participants assessed the relationship between atrial fibrillation (AF) and dementia risk [67,69,70,71]. The meta-analysis showed that AF was associated with an increased risk of dementia (pooled HR = 1.30 (95% CI: 1.12 to 1.50), I^2^ = 68.8%, low certainty) (Figure 7). Two studies, comprising a total of 251,997 participants, examined the association between AF and dementia subtypes. The pooled analysis indicated AF was significantly associated with a higher risk of AD (pooled HR = 1.29; 95% CI: 1.01 to 1.65; I^2^ = 0%, low certainty) (Supplementary Figure S4C). For vascular dementia, a pooled analysis could not be conducted due to the limited number of eligible studies. However, a single study reported that AF was associated with a substantially increased risk of vascular dementia (HR = 3.75; 95% CI: 2.56 to 5.50) [71]. Two studies reported results stratified by sex [67,71]. In the pooled analysis, AF was associated with a significantly increased risk of dementia among women (pooled HR = 1.21; 95% CI: 1.12–1.30, I^2^ = 3.6%). In contrast, no statistically significant association was observed for men (Supplementary Figure S4D).

### 3.4. Combined Effects of Obesity and CVDs on the Risk of Dementia

To evaluate the combined impact of obesity and CVD on dementia risk, a meta-analysis was not conducted due to the limited number of available studies. One study, involving 464,616 participants, examined the joint effects of BMI-defined obesity and CVD on all-cause dementia, with results reported separately for men and women [66]. The findings showed that men with obesity (BMI ≥ 30 kg/m^2^) and CVD had a 58% higher risk of all-cause dementia compared to men of normal weight without CVD (HR = 1.58; 95% CI: 1.37–1.81). Similarly, women with obesity and CVD had a 38% higher risk of all-cause dementia than men of normal weight without CVD (HR = 1.38; 95% CI: 1.16–1.64). Conversely, women of normal weight without CVD had a lower risk of all-cause dementia compared to men of normal weight without CVD (HR = 0.70; 95% CI: 0.63–0.77).

When examining dementia subtypes, men with obesity and CVD showed no significant association with Alzheimer’s disease (AD) risk (HR = 1.02; 95% CI: 0.80–1.30) compared with healthy-weight men without CVD. In contrast, obese women with CVD had a 37% higher risk of AD (HR = 1.37; 95% CI: 1.05–1.78). For vascular dementia, men with obesity and CVD had over three times the risk compared with healthy-weight men without CVD (HR = 3.14; 95% CI: 2.40–4.12), while women with obesity and CVD had a 74% higher risk relative to the same reference group (HR = 1.74; 95% CI: 1.22–2.50).

### 3.5. Quality Assessment

Overall, the included studies were of high quality based on their NOS assessment scores of 7 to 9. Of the 25 studies, three scored 7 (12%), ten scored 8 (40%), and the remaining twelve scored 9 (48%). The details of the quality assessment are shown in Supplementary Table S4.

### 3.6. Certainty of Evidence

The certainty of evidence for all outcomes was assessed using the GRADE approach based on prospective cohort studies (Table S5). As these were observational studies, all outcomes were initially rated as low certainty. Specifically, the evidence linking BMI-defined obesity to cognitive impairment was rated as low certainty. Despite no serious methodological limitations, certainty remained low due to the observational study design, which reflects limited confidence in estimated effects and suggests cautious interpretation.

Evidence regarding the association between BMI-defined obesity and the risk of dementia ranged from low to very low certainty. Downgrading was primarily driven by concerns related to inconsistency, as indicated by substantial heterogeneity across studies, and imprecision, given that pooled estimates showed no statistically significant association and the 95% confidence intervals crossed unity. The observed variability among studies further underscores the uncertainty surrounding these findings. The certainty of evidence for the association between central obesity and dementia risk was rated as low. Although the overall analysis showed considerable heterogeneity among studies, the subgroup analysis explained a substantial share of this variability.

The certainty of evidence for associations between CVDs and dementia risk was generally rated as low. For instance, the association between CHD and dementia risk was rated as low certainty, with no serious concerns identified across the GRADE domains of risk of bias, inconsistency, indirectness, or imprecision. Similarly, the certainty of evidence for the association between stroke and dementia risk was also rated as low. Detailed GRADE assessments for each outcome are provided in Supplementary Table S5.

## 4. Discussion

### 4.1. Main Findings

A total of twenty-five prospective cohort studies were included in the present study. Of these, 16 studies (64%) explored the link between BMI-defined obesity, central obesity (measured by waist circumference), and the risk of cognitive impairment or dementia. Eight studies (32%) focused on the relationship between CVDs and the risk of these conditions, and one study (4%) examined the combined effects of BMI-defined obesity and CVD on the risk of all-cause dementia.

Our meta-analysis of four prospective cohort studies demonstrated an inverse association between BMI-defined obesity and the risk of cognitive impairment. This finding may partly reflect reverse causality, particularly among older participants, as preclinical cognitive decline is often accompanied by weight loss, as well as the limited ability of BMI to capture age-related changes in body composition and fat distribution. Our finding is consistent with a longitudinal study of 2942 participants, which reported a 4% reduction in the odds of cognitive impairment among individuals with obesity (OR = 0.96; 95% CI: 0.93–0.99), irrespective of the analytical approach used [72]. Similarly, a study of 5239 adults aged 65 years and older found that BMI, after adjustment for covariates including waist circumference, was significantly associated with a lower risk of cognitive decline (HR = 0.97; 95% CI: 0.95–0.99) [73]. In contrast, a longitudinal cohort study of 7885 participants reported no significant association between BMI-defined obesity and cognitive impairment (RRR = 0.87; 95% CI: 0.67–1.14) [74]. This study was excluded from our analysis due to methodological limitations, as the association was examined using relative risk ratios (RRR) derived from multinomial logistic regression, an approach that does not adequately account for time-dependent factors. In our analysis, we considered a model in studies with fully adjusted covariates, including age, sex, type of residence, marital status, education, living arrangement, smoking status, drinking status, regular exercise, vegetable intake, and fruit intake. However, in the included studies related to cognitive impairment, average follow-up years varied from 4.07 to 12 years. Only one study had an average follow-up duration of 12 years, and the other three studies had follow-up durations of less than 10 years. The variability in follow-up durations warrants further research. Therefore, long-term follow-ups, more precise measurements of obesity, and larger and more diverse cohorts are essential for confirming these results and understanding the full impact of obesity on cognitive function.

The results of our meta-analysis indicated that obesity, as defined by BMI, did not exhibit a statistically significant association with the risk of dementia. This outcome aligns with prior meta-analysis, which similarly reported no significant relationship between BMI-defined obesity and dementia risk (pooled RR = 1.42; 95% CI: 0.93–2.18) when compared with individuals of normal BMI [75]. In contrast, findings from our random-effects meta-analysis indicated that experiencing obesity in midlife was associated with a 14% higher likelihood of developing dementia later in life (HR = 1.141; 95% CI: 1.004–1.292, I^2^ = 87%). This finding aligns with a previously conducted meta-analysis [47], which showed individuals classified as obese in midlife according to BMI exhibited a 47% higher risk of dementia (pooled RR = 1.47; 95% CI: 1.06–2.03; I^2^ = 59.9%). These findings indicate that being obese during midlife could raise the likelihood of developing dementia in later years, possibly because of lasting metabolic changes that promote neurodegeneration. In contrast, this association did not occur in older age, which may reflect shifts in how obesity influences health or the presence of other competing risk factors. These results highlight that weight reduction may have differential implications across the life course: maintaining a healthy weight during midlife may contribute to dementia prevention, whereas significant weight loss in late life may indicate underlying cognitive decline rather than confer protective effects.

Our analysis revealed that central obesity, measured by WC, was linked to a 14% increased risk of dementia. This finding agrees with evidence from a meta-analysis [27] showing that elevated WC was significantly associated with cognitive impairment and dementia (pooled HR = 1.10; 95% CI: 1.05–1.15, I^2^ = 86.8%). Similarly, our analysis showed that individuals with obesity, defined by waist circumference (WC) in late life, had a 13% greater risk of developing dementia (pooled HR = 1.13; 95% CI: 1.04–1.23; I^2^ = 36%). This outcome is consistent with the meta-analysis by Tang et al. (2021), which demonstrated that elevated WC in older adults (≥65 years) was significantly linked to both cognitive decline and dementia [27]. Collectively, these findings indicate that, among older populations, WC may serve as a more accurate anthropometric measure than BMI for evaluating obesity-related dementia risk. Our subgroup analysis revealed that, in women, obesity defined by WC was linked to a 24% higher risk of dementia. This result aligns with prior meta-analysis evidence showing that elevated WC was significantly associated with cognitive decline and dementia among women [27]. Women may be at higher risk due to hormonal changes after menopause and increased accumulation of visceral fat, which heightens metabolic and vascular risks. These factors are likely to strengthen the association between central obesity and dementia in women more than in men. Additionally, the sub-analysis showed that central obesity, defined by WC, was associated with a 28% higher risk of vascular dementia (pooled HR = 1.28; 95% CI: 1.01–1.62, I^2^ = 89%), while no link was observed with Alzheimer’s disease. This finding underscores the role of central obesity in cerebrovascular damage. However, the certainty of the evidence was low due to study design limitations and substantial heterogeneity among the included studies. The heterogeneity observed in the association between central obesity and dementia risk may, in part, be explained by variability in dementia ascertainment across studies. Diagnostic approaches differ widely, ranging from screening-based neuropsychological assessments to clinically or registry-based diagnoses. In addition, many of the included studies rely on administrative data and ICD codes to classify Alzheimer’s disease or vascular dementia, with variations across ICD versions and healthcare systems. This limitation may be particularly relevant for vascular cognitive impairments, where mixed pathology with coexisting Alzheimer’s disease is common. Such variability in outcome definition may contribute to the high between-study heterogeneity and, consequently, to the low certainty of evidence observed for the association between central obesity and incident dementia.

Regarding the relationship between CVDs and the risk of cognitive impairment or dementia, our analysis indicated mixed but informative findings. Four pooled studies demonstrated a strong association between stroke and an increased risk of dementia, highlighting the critical role of cerebrovascular events in cognitive decline. In contrast, two pooled studies reported that stroke was not significantly associated with the risk of cognitive impairment. The evidence indicates that stroke may play a contributory role in the development of sustained vascular injury and subsequent neurodegenerative processes. These pathological changes may not be immediately apparent as cognitive impairment (MCI) but have the potential to evolve into dementia over time [76]. Consistently, a meta-analysis [19] by Kuzma et al. (2018) reported that stroke was linked to a 69% increased risk of dementia (pooled HR = 1.69; 95% CI: 1.49–1.92; I^2^ = 87%). In addition, our meta-analysis demonstrated that CHD was associated with a 41% increased risk of dementia. Subgroup analyses further revealed significant associations between CHD and both Alzheimer’s disease (pooled HR = 1.37; 95% CI: 1.15–1.62, I^2^ = 0%) and vascular dementia (pooled HR = 2.06; 95% CI: 1.03–4.13, I^2^ = 91%), highlighting the heterogeneous influence of CHD across dementia subtypes. These findings are consistent with prior meta-analyses [77,78], one of which reported that a history of CHD conferred a 27% increased risk of dementia (pooled RR = 1.27; 95% CI: 1.07–1.50, I^2^ = 80%), while another identified a 22% increased risk specifically for Alzheimer’s disease. These findings reinforce the importance of cardiovascular health in the prevention of cognitive decline and suggest that managing CHD may play a crucial role in reducing the burden of dementia. Our analysis further identified a robust association between AF and an increased risk of dementia, with AF conferring approximately a 30% higher risk. Subgroup analyses demonstrated that AF was significantly related to AD (pooled HR = 1.29; 95% CI: 1.01–1.65, I^2^ = 0%), underscoring its potential role in neurodegenerative pathways.

In evaluating the combined effects of obesity and CVD on the risk of cognitive impairment and dementia, our systematic review identified only one prospective cohort study that examined the joint impact of BMI-defined obesity and CVD on all-cause dementia risk. The study presented results stratified by sex. It found that men with both obesity and CVD had a 58% higher risk of developing all-cause dementia compared to those without either condition. This finding indicates that when obesity coexists with vascular pathology, the detrimental effects dominate, substantially increasing dementia risk. The same study reported that men with obesity, but no CVD, had a 26% lower risk of all-cause dementia compared to men with normal BMI and no CVD. This suggests a possible obesity paradox because, in certain contexts, higher BMI may be protective against dementia risk, particularly in the absence of vascular disease. For women, having both obesity and CVD was linked to a 38% increased risk of all-cause dementia compared to men with a normal BMI and no CVD. In subgroup analyses, the study reported that men with both obesity and CVD had more than three times the risk of vascular dementia, although no significant link was found with Alzheimer’s disease. Conversely, women with obesity and CVD had a 37% and 74% higher risk of Alzheimer’s disease and vascular dementia, respectively, compared to men with a normal BMI and no CVD. Obesity constitutes a significant risk factor for cardiovascular disease (CVD) and, in conjunction with cerebrovascular small vessel disease (cSVD), represents a prevalent contributor to cognitive decline and vascular dementia [79]. Although obesity frequently co-occurs with CVD, both of which are strongly associated with heightened dementia risk, the literature reveals a notable gap concerning their combined influence on dementia outcomes.

From a clinical perspective, our findings suggest that individuals with central obesity, especially those over 60 years of age and those with coexisting cardiovascular disease, could represent a significant target group for dementia prevention efforts. In routine clinical practice, waist circumference measurements may provide additional information beyond BMI for identifying individuals at increased risk, particularly those suffering from vascular dementia. In midlife, lifestyle interventions aimed at reducing , BMI-defined obesity, and optimizing cardiovascular health may be particularly beneficial, since BMI-defined obesity during midlife is linked to an increased risk of dementia later in life.

### 4.2. Mechanisms

The present meta-analysis revealed an inverse association between BMI-defined obesity and cognitive impairment, thereby reinforcing evidence for the so-called “*obesity paradox*” in cognitive function among adults aged 40 years and older at baseline. This paradox describes the unexpected observation that obesity may exert protective effects against certain adverse health outcomes, including cognitive decline, in later life. Several mechanisms may account for this relationship. In older adults, BMI-defined obesity often reflects increased fat deposition in the lower extremities, and greater leg fat mass has been linked to improved glucose metabolism, which reduces the risk of cognitive impairment [80,81]. Moreover, higher BMI may signify enhanced energy reserves, which can mitigate age-related frailty and neurodegenerative processes [82]. Sufficient fat and muscle mass may also contribute to metabolic stability, thereby reducing susceptibility to cognitive deterioration. From a hormonal and metabolic perspective, adipose tissue functions as an active endocrine organ rather than a passive energy store [72,83]. It secretes leptin, a hormone with demonstrated neuroprotective properties that may enhance cognitive performance. Given the hippocampus’s central role in learning and memory, leptin’s modulatory effects on hippocampal function may partially explain its protective influence on cognitive outcomes [84].

Our meta-analysis demonstrated that central obesity, measured by WC, was significantly associated with an increased risk of dementia. One of the potential mechanisms underlying the relationship between central obesity and risk of cognitive decline is the activation of systemic and neuroinflammatory pathways [85]. Visceral adipose tissue, characterized by high metabolic activity, secretes pro-inflammatory cytokines that can traverse the blood–brain barrier. Once within the central nervous system, these mediators exacerbate neuroinflammation, thereby disrupting neuronal integrity and cognitive processes, which may ultimately contribute to the development of dementia [85]. Another plausible mechanism involves the interplay of oxidative stress, mitochondrial dysfunction, and vascular pathways. From the perspective of oxidative stress and mitochondrial impairment, excessive visceral adiposity heightens oxidative damage, leading to neuronal injury and disruption of neurovascular coupling [85]. With respect to vascular pathways, central obesity contributes to the progression of atherosclerosis, hypertension, and small vessel disease, conditions that compromise cerebral perfusion and thereby increase the risk of vascular dementia [86,87,88]. The strong association between central obesity and incident dementia is therefore likely to be explained by common pathophysiological mechanisms, including insulin resistance, chronic inflammation, endothelial dysfunction, and cerebral small vessel disease, all of which have a preferential impact on vascular cognitive pathways. A longitudinal study incorporating repeated measures of adiposity, as well as biomarkers and imaging-based assessments of neurodegeneration and cerebrovascular pathology, may help disentangle causal relationships, clarify the temporal sequence of weight change and cognitive decline, and improve risk stratification over the lifespan.

In our analysis, CVDs such as CHD, stroke, and AF were all found to be strongly linked to an increased risk of dementia. Notably, CHD showed a strong association with both AD and vascular dementia as well. The relationship between CVDs (CHD, stroke, and AF) and dementia risk may be explained by several underlying potential mechanisms. CHD leads to chronic hypoxic stress, white matter damage, and executive dysfunction, while heart failure contributes to neurohormonal and inflammatory stress, which accelerates cognitive decline [89]. In a stroke, ischemic or hemorrhagic lesions disrupt neural networks and cause strategic infarctions in regions such as the thalamus, hippocampus, and frontal-subcortical circuits, which cause disproportionate cognitive impairment [90,91]. AF increases the risk of clinical and subclinical embolism, resulting in cumulative microinfarcts and diffuse white matter damage [91]. Collectively, these mechanisms, which include hypoperfusion, infarctions, network disconnection, and systemic inflammation, may contribute to both vascular dementia and mixed dementia and may also interact with AD.

Coexistence of obesity and cardiovascular disease (CVD) may exacerbate dementia risk through several interrelated mechanisms [92]. The combined presence of obesity and CVD leads to oxidative stress, which in turn leads to the generation of free radicals that damage neurons and synaptic integrity. As a result of this dual burden, both metabolic and inflammatory disturbances are exacerbated: obesity contributes to inflammatory disruptions, while CVD leads to vascular damage. Together, these processes accelerate neurodegenerative changes to a greater extent than either condition in isolation.

## 5. Limitations of the Study

It is important to recognize the limitations of the present work. First, we did not evaluate the publication bias due to the limited number of included studies in the meta-analysis [42,48]. Although many of the included studies had large sample sizes and demonstrated high methodological quality, we still cannot exclude the possibility of publication bias. Second, the certainty of the evidence was limited by substantial heterogeneity and inconsistent reporting across some outcomes. Although we conducted subgroup analyses to explore possible contributors to this heterogeneity, its underlying causes remain uncertain. While low-certainty ratings largely reflected the reliance on study designs, very low certainty was driven by marked inconsistency and imprecision in effect estimates. These issues highlight the need for cautious interpretation of the findings. Third, we were unable to conduct a pooled analysis examining the combined impact of obesity and CVD on dementia risk due to the limited number of studies. To address this limitation, future research should employ longitudinal cohort designs incorporating baseline assessments and rigorously defined exposure measures. Such methodological approaches will enhance the identification of high-risk populations and support the development of more targeted preventive interventions.

## 6. Conclusions

In summary, the findings from our meta-analysis indicate that central obesity, measured by WC, is significantly associated with an increased risk of developing dementia, especially among individuals aged 60 and older. In contrast, obesity, defined by BMI, showed an inverse relationship with cognitive impairment. Although BMI-defined obesity was not directly linked to dementia risk, it was found to slightly increase the risk of dementia later in life when obesity occurs during midlife. CHD, stroke, and AF were strongly associated with dementia risk, with CHD being particularly linked to both AD and vascular dementia. Additionally, men with both obesity and CVD had a significantly higher risk of developing all-cause dementia, particularly a more than threefold increased risk of vascular dementia, although this finding is based on a single study. These findings highlight the necessity for future research to investigate the combined effects of obesity and CVD to address this gap in the existing evidence.

## Figures and Tables

**Table 1:** Characteristics of included studies.

First Author	Publication Year	Country	Study Design	Sample	Mean (SD) Age at Baseline	Sex (% Men)	Exposure and Ascertainment	Outcome and Ascertainment	Follow-Up (Years)	Outcome (N)	Covariates
Wu [50]	2021	China	Prospective cohort	12,027	81.23 (10.72)	47.48	BMI-defined obesity (≥28 kg/m^2^, Chinese guideline). Anthropometrics were measured by trained medical staff using standardized protocols.	Cognitive impairment. It was measured using the Chinese MMSE in home interviews; the scale is validated and reliable.	5.9 (2.8)	3086	Age, sex, type of residence, marital status, education, living arrangement, smoking status, drinking status, regular exercise, vegetables, and fruit intake
Liu [51]	2022	USA	Prospective cohort	10,798	58.3 (8.7)	41.2	BMI-defined obesity (≥30 kg/m^2^, WHO guideline). BMI calculated from self-reported height and weight.	Cognitive impairment was assessed using the Telephone Interview for Cognitive Status (TICS), a validated cognitive screening tool.	12 (6–16)	3297	Smoking, drinking, physical activity, baseline age, sex, education, marital status, PRS, and family wealth per capita
Liang [52]	2022	China	Prospective cohort	4792	80.70 (9.58)	50.61	BMI-defined obesity (≥28 kg/m^2^, Chinese guideline). Weight and height were measured by trained assessors using standardized procedures.	Cognitive impairment was assessed using the Chinese MMSE to evaluate cognitive function.	5	1077	Sex, age, residence, education, occupation, smoking status, alcohol consumption, regular exercise, financial independence, and health conditions.
Yang [53]	2025	South Korea	Prospective cohort	964,536	49.3 (6.0)	Women	BMI-defined obesity (≥25 kg/m^2^, Asia-Pacific WHO guideline) and abdominal obesity (WC ≥ 85 cm, Korean Society for the Study of Obesity guideline). Weight, height, and waist circumference were measured by trained examiners during health check-ups.	ACD, AD, and VaD were diagnosed using ICD-10 codes plus ≥2 prescriptions for anti-dementia medications. Subtypes: AD (ICD10: F00/G30) and VaD (F01).	8.2 (8–8.5)	ACD = 4495 AD = 3038 VaD = 823	age, smoking status, alcohol consumption, regular exercise, low household income, diabetes mellitus, hypertension, dyslipidaemia, depression, atrial fibrillation, stroke
Gong [54]	2021	UK	Prospective cohort	502,226	56.7 (8.2)	45.6	BMI-defined obesity (≥30 kg/m^2^, WHO guideline). BMI calculated from weight measured with a Tanita BC-418 MA body composition analyzer and standing height in meters.	All-cause dementia (ACD) diagnosed using ICD-10 codes (A81.0, F00, F01, F02, F03, F05, G30, G31.0, G31.1, G31.8, I67.3). Subtypes: AD (F00, G30) and VaD (F01, I67.3).	11.8	ACD = 4068	Age, SBP, diabetes, socioeconomic status, total Cholesterol, smoking status, lipid-lowering drugs, and antihypertensive drugs
Neergaard [55]	2016	Denmark	Prospective cohort	5512	70.7 (6.5)	Women	BMI-defined obesity (≥30 kg/m^2^, WHO guideline). Weight and height were measured.	ACD, AD, and VaD were diagnosed using ICD-10 codes (F02–F03 and R54). Subtypes: AD (F00, G30—G32) and VaD (F01).	11.9 (3.9)	ACD = 592 AD = 250 VaD = 43	Age, education, smoking, alcohol, physical activity, vascular, and neural disorders.
Cho [26]	2019	South Korea	Prospective cohort	872,082	70.2 (4.5)	45.6	BMI-defined obesity (≥25 kg/m^2^, Asian populations guideline) and abdominal obesity (WC ≥ 85 cm for women and ≥90 cm for men). Weight, height, and waist circumference were measured.	Dementia diagnosed using ICD-10 codes (F00, F01, F03, G30, and G318).	6.47	114,024	age; alcohol consumption; smoking and exercise status; systolic BP; FBS; HDL-C; LDL-C; AST; ALT; economic status; history of diabetes, hypertension, CVD, and CCI. Additionally, WC was adjusted for BMI, and BMI was adjusted for WC.
Ma [56]	2020	UK	Longitudinal study	6582	62.6 (9.0)	46.0	BMI-defined obesity (≥30 kg/m^2^, WHO guideline) and abdominal obesity (WC > 88 cm for women and >102 cm for men, NHLBI clinical guideline). Anthropometric measurements were taken by trained staff.	Dementia. Physicians diagnosed dementia in participants personally involved in the study, and secondly, dementia was diagnosed using a short-form informant questionnaire on cognitive decline.	11.4 (3.3)	453	age, sex, APOE E4, education, marital status, smoking status, physical activity, hypertension, and diabetes.
Tashiro [57]	2023	Japan	Prospective cohort	37,414	56.5 (7.8)	46.9	BMI-defined obesity (≥27 kg/m^2^, JPHC-based BMI categorization). BMI was calculated based on the self-reported weight and height of the participants.	Dementia was assessed using long-term care insurance (LTCI) certifications.	9.7	3019	Age, public health center area, smoking, alcohol drinking habits, hypertension, ischemic heart diseases, diabetes, cancer, and gastrointestinal ulcer.
Zhai [58]	2025	USA	Longitudinal study	23,255	72 (65–78)	43.4	BMI-defined obesity (≥30 kg/m^2^, WHO guideline). Weight and height were measured.	Cognitive impairment and dementia were assessed with MMSE, MoCA, digit span forward trials, verbal fluency, the Trail Making Test, WAIS-R Digit Symbol, the Boston Naming Test, NPI, and CDR.	4.07	CI = 1051 DM = 5968	age, gender, race, primary language, marital status, living situation, level of independence, handedness, smoking history, drinking history, APOE gene types, cancer, diabetes, heart disease, hypertension, hypercholesterolemia, vitamin B12 deficiency, and sleep disorders.
Yokomichi [59]	2020	Japan	Prospective cohort	3696	73.4 (5.8)	42.8	BMI-defined obesity (≥25 kg/m^2^, Asian populations guideline). Anthropometric measurements were taken by medical staff.	Dementia was assessed using long-term care insurance (LTCI) certifications.	5.8 (1.3)	338	Age, history of stroke, educational background, income, number of family members, marital status, and frequency of meeting friends.
Gottesman [60]	2017	USA	Prospective cohort	15,407	54.2 (5.8)	45	BMI-defined obesity (≥30 kg/m^2^, WHO guideline). The weight and height of the participants were taken by trained staff using standardized protocols.	Dementia was assessed using cognitive testing, neuropsychological evaluations, informant interviews, and telephone assessments, as well as hospital or death records.	23	1516	Age, sex, race, educational attainment, APOE ε4 genotype, diabetes, blood pressure status, and Hypercholesterolemia.
Chen [61]	2024	UK	Prospective cohort	466,788	56.8 (8.1)	46.4	WC-defined obesity (WC ≥ 94 cm for men and ≥80 cm for women, IDF European guideline). Waist circumference was measured by trained staff using standardized protocols.	Dementia was diagnosed using ICD-10 codes (F00–03).	12.7	6845	age, gender, UK Biobank assessment center, race, index of multiple deprivation, smoking status, alcohol consumption, physical activity, portions of fruit, vegetable intake, regular medications, mineral supplements, non-steroidal anti-inflammatory drugs, aspirin, and a history of Alzheimer’s disease/dementia
Ng [62]	2016	Singapore	Longitudinal cohort	1519	64.9 (6.8)	35.2	WC-defined obesity (WC ≥ 90 cm for men and ≥80 cm for women, IDF Asian populations guideline). Waist circumference was measured by trained staff using standardized protocols.	Cognitive impairment was assessed using the Informant Questionnaire on Cognitive Decline in the Elderly, the MMSE, and neurocognitive tests.	3.8	141	sex, age, education, APOE-ε4 genotype, smoking, and scores for physical, social, and productive activities.
Machado-Fragua [63]	2022	UK	Prospective cohort	7265	55.1 (2.9)	69.5	WC-defined obesity (WC ≥ 102 cm for men and ≥88 cm for women, WHO guideline). Waist circumference was measured.	Dementia was diagnosed using ICD-10 codes (F00–F03, F05.1, G30, and G31).	19.6 (5.9)	393	sex, education, ethnicity, birth cohort, smoking, alcohol consumption, consumption of fruits and vegetables, and physical activity.
Lee [64]	2020	South Korea	Prospective cohort	4,106,590	55.8 (10.1)	54.5	WC-defined obesity (WC ≥ 85 cm for women and ≥90 cm for men, Korean Society for the Study of Obesity guideline). Anthropometric measurements were taken by trained staff using standardized protocols.	Dementia was defined as having ≥2 prescriptions for anti-dementia medications together with an ICD-10 code for AD (F00 or G30), for VaD (F01), and other dementia (F02, F03, G23.1 or G31).	4.9	~77,953	age, sex, smoking, alcohol, regular exercise, stroke, depression, and CKD
Xiong [65]	2023	UK	Prospective cohort	171,538	64.1 (2.8)	48.5	CVDs (CHD and stroke) were assessed at baseline using self-reported diagnoses, hospital inpatient records, and ICD-10 codes (CHD: I20–I25; stroke: I60–I64).	Dementia was ascertained using hospital admission records, death registry data, and ICD-9 and 10 codes.	12.3 (11.5–13.0)	4479	Age, sex, ethnicity, education, socioeconomic status, deprivation, depression, APOE ε4, cognition, lifestyle category
Dong [66]	2022	UK	Prospective cohort	464,616	56.6 (8.1)	46	CVDs (CHD, stroke, and HF) were ascertained from hospital medical records along with ICD-9 and ICD-10 codes. BMI-defined obesity (≥30 kg/m^2^, WHO guideline)	Dementia was assessed using ICD-9 and 10 codes. ICD codes (F00, F01, F02, F03, G30, G31·0, G31·8) and ICD-9 code (290·1).	11.2 (1.5)	5527	age, race/ethnicity, educational years, income level, physical activity level, leisure activities, body mass index (BMI), smoking status, diabetes status, hypertension status, and APOE
Boivin-Proulx [67]	2023	Canada	Prospective cohort	320,630	74.1 (6.5)	42.3	CVDs (HF, stroke, and AF) were ascertained using ICD-9 and ICD-10 codes.	Dementia was diagnosed using ICD-9 codes (46.1, 331.0, 331.1, 331.5, 290, 294), ICD-10 codes (G30, F00–F03), and prescriptions for anti-dementia medications.	4 (2–5)	30,626	age, sex, all cardiovascular risk factors or diseases, including major bleeding, systemic embolism, peripheral artery disease, and chronic kidney disease.
Dove [32]	2022	Sweden	Longitudinal study	1873	72.4 (10.0)	38.1	CVDs (stroke and CHD) were assessed using clinical examinations, medical history, and medical records.	Cognitive impairment was assessed using a battery of neuropsychological tests, perceptual Speed, verbal fluency, and semantic memory.	11.2	539	Baseline age, sex, education, body mass index (BMI), physical activity, hypertension, alcohol consumption, and APOE ԑ4 carrier status.
Dove [31]	2023	Sweden	Prospective cohort	17,913	70.1 (7.5)	45	CVDs (stroke and CHD) were assessed using ICD-7, 8, 9, 10 codes.	ACD, AD, and VaD were diagnosed based on records from the NPR and using ICD-8, 9, and 10 codes.	15.4	ACD = 3020 AD = 1050 VaD = 638	age, sex, education level, marital status, BMI, hypertension, smoking status, alcohol consumption, physical activity level, and depression.
Wu [68]	2021	USA	Prospective cohort	5290	75.3 (7.4)	43.4	Stroke was assessed through self-reports by participants if they had been diagnosed with stroke by physicians.	Cognitive impairment was determined using three neurocognitive tests, including memory, orientation, and executive functioning.	8	1458	age, sex, race/ethnicity, educational level, Medicare–Medicaid eligibility, proxy respondent, depression, anxiety, smoking status, comorbidities, and BMI
De Bruijn [69]	2015	Netherlands	Prospective cohort	6514	≥55 *	44	AF was diagnosed using ECGs, and the results were confirmed by physicians.	Dementia was assessed using MMSE and the Geriatric Mental State Schedule organic level.	12.5	DM = 994 AD = 787	Age, sex, diabetes mellitus, smoking, total cholesterol and high-density lipoprotein cholesterol levels, lipid-lowering medication, systolic and diastolic blood pressure, blood pressure–lowering medication, body mass index, educational level, ever use of oral anticoagulant medication, coronary heart disease, heart failure, and apolipoprotein E ε4 carrier status.
Imahori [70]	2023	Sweden	Prospective cohort	2568	72.3 (9.9)	38.1	IHD and AF were diagnosed by clinical examination of physicians, NPR using ICD-10 codes.	Dementia was diagnosed using DSM-IV criteria.	11.4 (7.1–11.7)	379	age, sex, and education, smoking, alcohol consumption, physical activity, body mass index, total cholesterol, hypertension, diabetes, apolipoprotein E (APOE) genotype, cerebrovascular diseases, chronic obstructive pulmonary disease, and C-reactive protein.
Hu [71]	2022	UK	Prospective cohort	245,483	62.32 (4)	46.8	CVDs (AF and IHD) were assessed using ICD-10 codes.	ACD, AD, and VaD were diagnosed according to the ICD-9 and 10 codes.	9.26 (7.15–10.78)	ACD = 5123 AD = 2228 VaD = 1234	age, sex, education, BMI, physical activity, smoking, and *APOE4* status

MMSE: mini-mental state examination, MoCA: Montreal Cognitive Assessment, CDR: clinical dementia rating, Swedish National Patient Register (NPR), ACD: all-cause dementia, *: baseline age rather than the mean age.

## Data Availability

The original contributions presented in this study are included in the article/Supplementary Materials. Further inquiries can be directed to the corresponding author.

## Supplementary Materials

The following supporting information can be downloaded at: https://www.mdpi.com/article/10.3390/ijms27041892/s1.
