# Supplementary material for: Independent and Combined Effects of Obesity and Cardiovascular Diseases on the Risk of Cognitive Impairment and Dementia: A Systematic Review and Meta-Analysis of Prospective Cohort Studies Involving 8,276,914 Participants
Source: Int J Mol Sci. 2026 Feb 16;27(4):1892. doi: 10.3390/ijms27041892 (PMC12940507; doi:10.3390/ijms27041892)
Supplement: Supplementary file 1 [file ijms-27-01892-s001.zip › Supplemental materials.pdf]

Supplemental materials

Independent and Combined Effects of Obesity and Cardiovascular Diseases on the Risk of Cognitive Impairment and Dementia: A Systematic Review and Meta-Analysis of Prospective Cohort Studies Involving 8,276,914 Participants

Getu Gamo Sagaro[1,2] and Seyed Khosrow Tayebati[1]

[1]School of Pharmaceutical and Health Products Sciences, University of Camerino, Camerino, Italy

[2]School of Public Health, College of Health Sciences and Medicine, Wolaita Sodo University, Sodo, Ethiopia.

Corresponding Authors:
Getu Gamo Sagaro, PhD, MPH
getugamo.sagaro@unicam.it
gamogetu48@gmail.com
cell phone: (+39) 3533012484

and
Seyed Khosrow Tayebati, PharmD, PhD, ClinPharmD
khosrow.tayebati@unicam.it
khosrow.tayebati@gmail.com
Phone: (+39) 0737403305

List of supplemental files

1. Table S1: List of search terms.
2. Table S2: PICOS criteria.
3. Table S3**:** Results of leave-one-out sensitivity analysis of the association between BMI-defined obesity and risk of dementia**.**
4. Table S4: Risk of bias assessment using the Newcastle-Ottawa Scale (NOS).
5. Table S5: Certainty of the evidence assessment using the Grading of Recommendations, Assessment, Development, and Evaluation (GRADE) approach.
6. Figure S1 A: Forest plot of the association between BMI-defined obesity and risk of dementia types.
7. Figure S1 B: Forest plot of the association between BMI-defined obesity and risk of dementia, stratified by gender (Men vs Women).
8. Figure S2 A: Forest plot of the association between BMI-defined obesity and risk of dementia, stratified by follow-up years (< 10 years vs ≥ 10 years).
9. Figure S2 B: Forest plot of the association between BMI-defined obesity and risk of dementia, stratified by BMI cut-off values (BMI ≥25 kg/m² vs BMI ≥30 kg/m²).
10. Figure S3 A: Forest plot of the association between WC-defined obesity and risk of dementia, stratified by gender (Men vs. Women).
11. Figure S3 B: Forest plot of the association between WC-defined obesity and risk of dementia, stratified by follow-up years (< 10 years vs ≥ 10 years).
12. Figure S4 A: Forest plot of the association between coronary heart disease (CHD) and risk of dementia, stratified by dementia types.
13. Figure S4 B: Forest plot of the association between stroke and risk of cognitive dementia.
14. Figure S4 C: Forest plot of the association between atrial fibrillation (AF) and risk of dementia, stratified by dementia types.
15. Figure S4 D: Forest plot of the association between AF and risk of dementia, stratified by sex (Men vs. Women).

.

Table S1: List of search terms.

Database	Objective	Search terms	Results
**PubMed**	#1	(obesity[MeSH Terms] OR obesity[tiab] OR obese[tiab] OR "body mass index"[tiab] OR BMI[tiab] OR "waist circumference"[tiab] OR WC[tiab] OR "waist-hip ratio"[tiab] OR WHR[tiab] OR "waist to hip ratio"[tiab] OR "waist-to-height ratio"[tiab] OR WHtR[tiab] OR "body fat percentage"[tiab] OR "body fat mass"[tiab] OR "fat mass"[tiab] OR "fat mass index"[tiab] OR FMI[tiab] OR adiposity[tiab] OR "central adiposity"[tiab] OR "visceral adipose tissue"[tiab] OR "subcutaneous fat"[tiab] OR "truncal fat"[tiab] OR "fat distribution"[tiab] OR "body composition"[tiab]) AND (cognition[MeSH Terms] OR cognition[tiab] OR "cognitive function"[tiab] OR "cognitive performance"[tiab] OR "cognitive impairment"[tiab] OR "cognitive decline"[tiab] OR "cognitive dysfunction"[tiab] OR neurocognitive disorders[MeSH Terms] OR Mild Cognitive Impairment[MeSH Terms] OR MCI[tiab] OR memory[MeSH Terms] OR memory[tiab] OR memory disorders[MeSH Terms] OR"executive function"[tiab] OR "processing speed"[tiab] OR "verbal fluency"[tiab] OR attention[tiab] OR language[tiab] OR "cognitive aging"[tiab] OR "cognitive reserve"[tiab] OR dementia[MeSH Terms] OR dementia[tiab] OR Alzheimer Disease[MeSH Terms] OR Alzheimer*[tiab]) AND ("cohort studies"[MeSH Terms] OR cohort[tiab] OR cohorts[tiab] OR "longitudinal studies"[MeSH Terms] OR longitudinal[tiab] OR prospective[tiab] OR prospectively[tiab])	9,739
**Scopus**	#1	(TITLE-ABS-KEY(obesity) OR TITLE-ABS-KEY(obese) OR TITLE-ABS-KEY("body mass index") OR TITLE-ABS-KEY(BMI) OR TITLE-ABS-KEY("waist circumference") OR TITLE-ABS-KEY(WC) OR TITLE-ABS-KEY("waist-hip ratio") OR TITLE-ABS-KEY(WHR) OR TITLE-ABS-KEY("waist to hip ratio") OR TITLE-ABS-KEY("waist-to-height ratio") OR TITLE-ABS-KEY(WHtR) OR TITLE-ABS-KEY("body fat percentage") OR TITLE-ABS-KEY("body fat mass") OR TITLE-ABS-KEY("fat mass") OR TITLE-ABS-KEY("fat mass index") OR TITLE-ABS-KEY(FMI) OR TITLE-ABS-KEY(adiposity) OR TITLE-ABS-KEY("central adiposity") OR TITLE-ABS-KEY("visceral adipose tissue") OR TITLE-ABS-KEY("subcutaneous fat") OR TITLE-ABS-KEY("truncal fat") OR TITLE-ABS-KEY("fat distribution") OR TITLE-ABS-KEY("body composition")) AND (TITLE-ABS-KEY(cognition) OR TITLE-ABS-KEY("cognitive function") OR TITLE-ABS-KEY("cognitive performance") OR TITLE-ABS-KEY("cognitive impairment") OR TITLE-ABS-KEY("cognitive decline") OR TITLE-ABS-KEY("cognitive dysfunction") OR TITLE-ABS-KEY(MCI) OR TITLE-ABS-KEY(memory) OR TITLE-ABS-KEY("memory disorders") OR TITLE-ABS-KEY("executive function") OR TITLE-ABS-KEY("processing speed") OR TITLE-ABS-KEY("verbal fluency") OR TITLE-ABS-KEY(attention) OR TITLE-ABS-KEY(language) OR TITLE-ABS-KEY("cognitive aging") OR TITLE-ABS-KEY("cognitive reserve") OR TITLE-ABS-KEY(dementia) OR TITLE-ABS-KEY("alzheimer disease") OR TITLE-ABS-KEY(alzheimer*)) AND (TITLE-ABS-KEY(cohort OR cohorts OR longitudinal OR "longitudinal study" OR "longitudinal studies" OR prospective OR prospectively))	10,797
**Web of Science**	#1	TS=(obesity OR obese OR "body mass index" OR BMI OR "waist circumference" OR WC OR "waist-hip ratio" OR WHR OR "waist to hip ratio" OR "waist-to-height ratio" OR WHtR OR "body fat percentage" OR "body fat mass" OR "fat mass" OR "fat mass index" OR FMI OR adiposity OR "central adiposity" OR "visceral adipose tissue" OR "subcutaneous fat" OR "truncal fat" OR "fat distribution" OR "body composition") AND TS=(cognition OR "cognitive function" OR "cognitive performance" OR "cognitive impairment" OR "cognitive decline" OR "cognitive dysfunction" OR MCI OR memory OR "memory disorders" OR "executive function" OR "processing speed" OR "verbal fluency" OR attention OR language OR "cognitive aging" OR	7154

		"cognitive reserve" OR dementia OR "alzheimer disease" OR alzheimer*) AND TS=(cohort OR cohorts OR "cohort study" OR "cohort studies" OR longitudinal OR "longitudinal study" OR "longitudinal studies" OR prospective OR prospectively)	
**PubMed**	#2	("cardiovascular diseases"[MeSH Terms] OR "cardiovascular disease"[tiab] OR CVD[tiab] OR"heart failure"[MeSH Terms] OR "heart failure"[tiab] OR "myocardial infarction"[MeSH Terms] OR "myocardial infarction"[tiab] OR MI[tiab] OR "ischemic heart disease"[tiab] OR "coronary heart disease"[tiab] OR CHD[tiab] OR "coronary artery disease"[tiab] OR CAD[tiab] OR "stroke"[MeSH Terms] OR "stroke"[tiab] OR "transient ischemic attack"[tiab] OR TIA[tiab] OR "cerebrovascular disorders"[MeSH Terms] OR cerebrovascular[tiab] OR "atherosclerosis"[MeSH Terms] OR atherosclerosis[tiab] OR "vascular disease"[tiab] OR "vascular disorders"[tiab] OR "peripheral artery disease"[tiab] OR PAD[tiab] OR "carotid artery disease"[tiab] OR "cardiovascular event"[tiab] OR "cardiovascular events"[tiab]) AND (cognition[MeSH Terms] OR cognition[tiab] OR "cognitive function"[tiab] OR "cognitive performance"[tiab] OR "cognitive impairment"[tiab] OR "cognitive decline"[tiab] OR "cognitive dysfunction"[tiab] OR neurocognitive disorders[MeSH Terms] OR Mild Cognitive Impairment[MeSH Terms] OR MCI[tiab] OR memory[MeSH Terms] OR memory[tiab] OR memory disorders[MeSH Terms] OR "executive function"[tiab] OR "processing speed"[tiab] OR "verbal fluency"[tiab] OR attention[tiab] OR language[tiab] OR "cognitive aging"[tiab] OR "cognitive reserve"[tiab] OR dementia[MeSH Terms] OR dementia[tiab] OR Alzheimer Disease[MeSH Terms] OR Alzheimer*[tiab]) AND ("cohort studies"[MeSH Terms] OR "cohort"[tiab] OR "cohorts"[tiab] OR "longitudinal studies"[MeSH Terms] OR longitudinal[tiab] OR prospective[tiab] OR prospectively[tiab])	17, 842
**Scopus**	#2	(TITLE-ABS-KEY("cardiovascular disease" OR CVD OR "heart failure" OR "myocardial infarction" OR MI OR "ischemic heart disease" OR "coronary heart disease" OR CHD OR "coronary artery disease" OR CAD OR stroke OR "transient ischemic attack" OR TIA OR cerebrovascular OR atherosclerosis OR "vascular disease" OR "vascular disorders" OR "peripheral artery disease" OR PAD OR "carotid artery disease" OR "cardiovascular event" OR "cardiovascular events")) AND (TITLE-ABS-KEY(cognition OR "cognitive function" OR "cognitive performance" OR "cognitive impairment" OR "cognitive decline" OR "cognitive dysfunction" OR MCI OR memory OR "memory disorders" OR "executive function" OR "processing speed" OR "verbal fluency" OR attention OR language OR "cognitive aging" OR "cognitive reserve" OR dementia OR "alzheimer disease" OR alzheimer*)) AND (TITLE-ABS-KEY(cohort OR cohorts OR "cohort study" OR "cohort studies" OR longitudinal OR "longitudinal study" OR "longitudinal studies" OR prospective OR prospectively))	22,972
**Web of Science**	#2	TS=("cardiovascular disease" OR CVD OR "heart failure" OR "myocardial infarction" OR MI OR "ischemic heart disease" OR "coronary heart disease" OR CHD OR "coronary artery disease" OR CAD OR stroke OR "transient ischemic attack" OR TIA OR cerebrovascular OR atherosclerosis OR "vascular disease" OR "vascular disorders" OR "peripheral artery disease" OR PAD OR "carotid artery disease" OR "cardiovascular event" OR "cardiovascular events") AND TS=(cognition OR "cognitive function" OR "cognitive performance" OR "cognitive impairment" OR "cognitive decline" OR "cognitive dysfunction" OR MCI OR memory OR "memory disorders" OR "executive function" OR "processing speed" OR "verbal fluency" OR attention OR language OR "cognitive aging" OR "cognitive reserve" OR dementia OR "alzheimer disease" OR alzheimer*) AND TS=(cohort OR cohorts OR "cohort study" OR "cohort studies" OR longitudinal OR "longitudinal study" OR "longitudinal studies" OR prospective OR prospectively)	13,711

**PubMed**	# 3	(obesity[MeSH Terms] OR obesity[tiab] OR obese[tiab] OR "body mass index"[tiab] OR BMI[tiab] OR "waist circumference"[tiab] OR WC[tiab] OR "waist-hip ratio"[tiab] OR WHR[tiab] OR "waist to hip ratio"[tiab] OR "waist-to-height ratio"[tiab] OR WHtR[tiab] OR "body fat percentage"[tiab] OR "body fat mass"[tiab] OR "fat mass"[tiab] OR "fat mass index"[tiab] OR FMI[tiab] OR adiposity[tiab] OR "central adiposity"[tiab] OR "visceral adipose tissue"[tiab] OR "subcutaneous fat"[tiab] OR "truncal fat"[tiab] OR "fat distribution"[tiab] OR "body composition"[tiab]) AND ("cardiovascular diseases"[MeSH Terms] OR "cardiovascular disease"[tiab] OR CVD[tiab] OR "heart failure"[MeSH Terms] OR "heart failure"[tiab] OR "myocardial infarction"[MeSH Terms] OR "myocardial infarction"[tiab] OR MI[tiab] OR "ischemic heart disease"[tiab] OR "coronary heart disease"[tiab] OR CHD[tiab] OR "coronary artery disease"[tiab] OR CAD[tiab] OR "stroke"[MeSH Terms] OR "stroke"[tiab] OR "transient ischemic attack"[tiab] OR TIA[tiab] OR "cerebrovascular disorders"[MeSH Terms] OR cerebrovascular[tiab] OR "atherosclerosis"[MeSH Terms] OR atherosclerosis[tiab] OR "vascular disease"[tiab] OR "vascular disorders"[tiab] OR "peripheral artery disease"[tiab] OR PAD[tiab] OR "carotid artery disease"[tiab] OR "cardiovascular event"[tiab] OR "cardiovascular events"[tiab]) AND (cognition[MeSH Terms] OR cognition[tiab] OR "cognitive function"[tiab] OR "cognitive performance"[tiab] OR "cognitive impairment"[tiab] OR "cognitive decline"[tiab] OR "cognitive dysfunction"[tiab] OR neurocognitive disorders[MeSH Terms] OR Mild Cognitive Impairment[MeSH Terms] OR MCI[tiab] OR memory[MeSH Terms] OR memory[tiab] OR memory disorders[MeSH Terms] OR "executive function"[tiab] OR "processing speed"[tiab] OR "verbal fluency"[tiab] OR attention[tiab] OR language[tiab] OR "cognitive aging"[tiab] OR "cognitive reserve"[tiab] OR dementia[MeSH Terms] OR dementia[tiab] OR Alzheimer Disease[MeSH Terms] OR Alzheimer*[tiab]) AND ("cohort studies"[MeSH Terms] OR cohort[tiab] OR cohorts[tiab] OR "longitudinal studies"[MeSH Terms] OR longitudinal[tiab] OR prospective[tiab] OR prospectively[tiab])	1,585
**Scopus**	# 3	(TITLE-ABS-KEY(obesity OR obese OR "body mass index" OR BMI OR "waist circumference" OR WC OR "waist-hip ratio" OR WHR OR "waist to hip ratio" OR "waist-to-height ratio" OR WHtR OR "body fat percentage" OR "body fat mass" OR "fat mass" OR "fat mass index" OR FMI OR adiposity OR "central adiposity" OR "visceral adipose tissue" OR "subcutaneous fat" OR "truncal fat" OR "fat distribution" OR "body composition"))<br>AND (TITLE-ABS-KEY("cardiovascular disease" OR CVD OR "heart failure" OR "myocardial infarction" OR MI OR "ischemic heart disease" OR "coronary heart disease" OR CHD OR "coronary artery disease" OR CAD OR stroke OR "transient ischemic attack" OR TIA OR cerebrovascular OR atherosclerosis OR "vascular disease" OR "vascular disorders" OR "peripheral artery disease" OR PAD OR "carotid artery disease" OR "cardiovascular event" OR "cardiovascular events")) AND (TITLE-ABS-KEY(cognition OR "cognitive function" OR "cognitive performance" OR "cognitive impairment" OR "cognitive decline" OR "cognitive dysfunction" OR MCI OR memory OR "memory disorders" OR "executive function" OR "processing speed" OR "verbal fluency" OR attention OR language OR "cognitive aging" OR "cognitive reserve" OR dementia OR "alzheimer disease" OR alzheimer*)) AND (TITLE-ABS-KEY(cohort OR cohorts OR "cohort study" OR "cohort studies" OR longitudinal OR "longitudinal study" OR "longitudinal studies" OR prospective OR prospectively))	3142
**Web of Science**	# 3	TS=(obesity OR obese OR "body mass index" OR BMI OR "waist circumference" OR WC OR "waist-hip ratio" OR WHR OR "waist to hip ratio" OR "waist-to-height ratio" OR WHtR OR<br>"body fat percentage" OR "body fat mass" OR "fat mass" OR "fat mass index" OR FMI OR adiposity OR "central adiposity" OR "visceral adipose tissue" OR "subcutaneous fat" OR "truncal fat" OR "fat distribution" OR "body composition") AND TS=("cardiovascular disease" OR CVD OR "heart failure" OR "myocardial infarction" OR MI OR "ischemic heart disease" OR "coronary heart disease" OR CHD OR "coronary artery disease" OR CAD OR stroke	1440

		OR "transient ischemic attack" OR TIA OR cerebrovascular OR atherosclerosis OR "vascular disease" OR "vascular disorders" OR "peripheral artery disease" OR PAD OR "carotid artery disease" OR "cardiovascular event" OR "cardiovascular events") AND TS=(cognition OR "cognitive function" OR "cognitive performance" OR "cognitive impairment" OR "cognitive decline" OR "cognitive dysfunction" OR MCI OR memory OR "memory disorders" OR "executive function" OR "processing speed" OR "verbal fluency" OR attention OR language OR "cognitive aging" OR "cognitive reserve" OR dementia OR "alzheimer disease" OR alzheimer*) AND TS=(cohort OR cohorts OR "cohort study" OR "cohort studies" OR longitudinal OR "longitudinal study" OR "longitudinal studies" OR prospective OR prospectively)	

Objective #1*: Search terms for the studies assessing the association between obesity and the risk of cognitive impairment and dementia.*

Objective #2*: Search terms for the studies examining the association between CVDs and the risk of cognitive impairment and dementia.*

Objective #3*: Search terms for the studies on the combined effects of obesity and CVDs on the risk of cognitive impairment and dementia.*

Supplemental **Table S2**: PICOS criteria

**Indicator**	**Inclusion**	**Exclusion**
Population (P)	Adults aged 40 years and older at baseline	Individuals younger than 40 years at baseline
Intervention/Exposure (I)	Obesity defined by BMI, WC, or WHR; presence of CVD; or both conditions at baseline	Studies that reported BMI, WC, or WHR only as continuous variables without categorical classification
Comparator (C)	Individuals with normal BMI, those in the lowest category of WC/WHR, non-CVD individuals, or those with either obesity or CVD alone, or neither condition.	Studies lacking an appropriate comparison group (e.g., normal weight or non-CVD)
Outcomes (O)	Incidence of cognitive impairment or dementia. Secondary outcomes: AD and VaD	Cognitive impairment assessed by imaging (e.g., MRI or CT)
Study design(S)	Prospective cohort studies or other longitudinal follow-up designs.	Cross-sectional studies, case-control studies, retrospective cohorts, clinical trials, experimental studies, reviews (systematic reviews, meta-analyses, narrative reviews), and case studies

Supplementary **Table S3:** Results of leave-one-out sensitivity analysis of the association between BMI-defined obesity and risk of dementia**.**

**Study Excluded**	**Pooled HR (95% CI)**	**Heterogeneity (I^2)**
Yang et al.,2025	0.98 [0.83 - 1.15]	95%
Gong et al.,2021	1.00 [0.85 - 1.18]	96.3%
Neergaard et al.,2016	1.02 [0.88 - 1.19]	96.7%
Cho et al.,2019	1.03 [0.88 - 1.20]	91%
Ma et al.,2020	0.97 [0.84 - 1.12]	96.5%
Tashiro et al.,2023	0.96 [0.84; 1.10]	95.5%
Zhai et al.,2025	1.03 [0.89; 1.20]	96.7%
Yokomichi et al.,2020	1.02 [0.87 - 1.18]	96.7%
Gottesman et al.,2017	0.98 [0.84 - 1.15]	96.4%

Supplemental **Table S4**: Risk of bias assessment using the Newcastle-Ottawa Scale (NOS).

**Study**	**Selection**				**Comparability**		**Outcome**			**Total**
	1	2	3	4	5	6	7	8	9	Quality score
Wu et al.,2021	*	*	*	*	*	*	*	*	*	**9**
Liu et al.,2022	*	*	-	*	*	*	*	*	*	**8**
Liang et al.,2022	*	*	*	*	*	*	*	-	*	**8**
Yang et al.,2025	*	*	*	*	*	*	*	*	*	**9**
Gong et al.,2021	*	*	*	*	*	-	*	*	*	**8**
Neergaard et al.,2016	-	*	*	*	*	*	*	*	*	**8**
Cho et al.,2019	*	*	*	*	*	*	*	*	*	**9**
Ma et al.,2020	*	*	*	*	*	*	*	*	*	**9**
Tashiro et al.,2023	*	*	-	*	*	-	*	*	*	**7**
Zhai et al.,2025	*	*	*	*	*	*	*	-	*	**8**
Yokomichi et al.,2020	*	*	*	*	*	-	*	*	*	**8**
Gottesman et al.,2017	*	*	*	*	*	*	*	*	*	**9**
Chen et al.,2024	*	*	*	*	*	-	*	*	*	**8**
Machado-Fragua *et al.*, 2022	*	*	*	*	*	*	*	*	*	**9**
Lee et al.,2020	*	*	*	*	*	*	*	-	*	**8**
Ng et al.,2016	*	*	*	-	*	*	*	-	*	**7**
Imahori et al.,2023	*	*	*	*	*	*	*	*	*	**9**
Dong et a.,2022	*	*	*	*	*	*	*	*	*	**9**
Dove et al.,2023	*	*	*	*	*	*	*	*	*	**9**

De Bruijn R et al.,2015	*	*	*	*	*	*	*	*	*	**9**
Wu et al.,2021	*	*	-	*	*	*	*	*	*	**8**
Xiong et al.,2023	*	*	-	*	*	*	*	*	*	**8**
Boivin-Proulx et al.,2023	*	*	*	*	*	-	*	-	*	**7**
Dove et al.,2022	*	*	*	*	*	*	*	*	*	**9**
Hu et al.,2022	*	*	*	*	*	*	*	*	*	**9**

A study may receive a maximum of one star for each numbered item within the category of Selection and Outcome. For Comparability, a maximum of two stars may be awarded.* ***For the observational studies**, selection: (1) representativeness ofthe exposed cohort, (2) selection of the non-exposed cohort, (3) ascertainment of exposure, and (4) demonstration that the outcome of interest was not present at the start of the study. Comparability: (5) study control for age and sex, (6) study control for additional factors.Outcome: (7) assessment of outcome, (8) follow-up long enough for outcomes to occur, (9) adequacy of follow-up.*

Supplemental **Table S5**: Certainty of the evidence assessment using the Grading of Recommendations, Assessment, Development, and Evaluation (GRADE) approach.

**Outcome**	**Exposure definition**	**Number of studies**	**Certainty assessment**						**Effect**	**Certainty of evidence‡**
			Risk of bias	Inconsistency	Indirectness	Imprecision	Other considerations			
							Publication bias	other	HR (95% CI)	
Cognitive impairment	Obesity (BMI)	4	not serious	not serious	not serious	not serious	undetected	none	0.85 (0.74 to 0.98)	Low
Dementia	Obesity (BMI)	9	not serious	serious[a]	not serious	serious[b]	undetected	none	1.00 (0.86 to 1.15)	very low
Alzheimer's disease	Obesity (BMI)	3	not serious	serious[c]	not serious	serious[d]	undetected	none	0.85 (0.65 to 1.12)	very low
Vascular Dementia	Obesity (BMI)	3	not serious	serious[e]	not serious	serious[f]	undetected	none	0.98 (0.90 to 1.45)	very low
Dementia	Central obesity (WC)	6	not serious	not serious[g]	not serious	not serious	undetected	none	1.14 (1.03 to 1.27)	Low
Alzheimer's disease	Central obesity (WC)	3	not serious	serious[h]	not serious	serious[i]	undetected	none	1.04 (0.85 to 1.28)	Very low
Vascular Dementia	Central obesity (WC)	3	not serious	serious[j]	not serious	not serious	undetected	none	1.28 (1.01 to 1.62)	Very low
Dementia	CHD	5	not serious	not serious	not serious	not serious	undetected	none	1.41 (1.29 to 1.54)	Low
Dementia	Stroke	4	not serious	not serious	not serious	not serious	undetected	none	1.53 (1.35 to 1.74)	Low
Dementia	Atrial Fibrillation	4	not serious	not serious	not serious	not serious	undetected	none	1.30 (1.12 to 1.50)	Low
Alzheimer's disease	CHD	2	not serious	not serious	not serious	not serious	undetected	none	1.37 (1.15 to 1.62)	Low
Vascular Dementia	CHD	2	not serious	Serious[k]	not serious	Serious[l]	undetected	none	2.06 (1.03 to 4.13)	Very low
Alzheimer's disease	Atrial Fibrillation	2	not serious	not serious	not serious	not serious	undetected	none	1.29 (1.01 to 1.65)	Low
Cognitive impairment	Stroke	2	not serious	not serious	not serious	serious[m]	undetected	none	1.06 (0.68 to 1.66)	Very low

BMI = body mass index, WC = waist circumference, CHD = coronary heart disease, and ‡ certainty of evidence starts Low due to observational study design.

Explanations

a. Downgraded by 1 level for inconsistency: Visual inconsistency and statistical analysis also showing heterogeneity (I^2 = 98.2%).

b. Downgraded by 1 level for imprecision: The 95% CI includes an HR of 1.

c. Downgraded by 1 level for inconsistency: visual inconsistency and statistical analysis also showing heterogeneity (I^2 = 94.3%).

d. Downgraded by 1 level for imprecision: The 95% CI (0.65 to 1.12) includes an HR of 1.

e. Downgraded by 1 level for inconsistency: Substantial heterogeneity was detected (I^2 = 84%), and the variability in effect estimates was not explained

f. Downgraded by 1 level for imprecision: The 95% CI crosses no effect (HR = 1).

g. Not downgraded by 1 level for inconsistency: Although considerable heterogeneity was detected (I^2 = 96.1%), the subgroup analysis explained a substantial share of this variability.

h. Downgraded by 1 level for inconsistency: Substantial heterogeneity was observed (I^2 = 94.5%).

i. Downgraded by 1 level for imprecision: The 95% CI (0.85 to 1.28) crosses no effect (HR = 1).

j. Downgraded by 1 level for inconsistency: Considerable heterogeneity was detected (I^2 = 89.4%).

k. Downgraded by 1 level for inconsistency: The statistical analysis showed considerable heterogeneity (I^2 = 90.9%).

l. Downgraded by 1 level for imprecision: The 95% CI (1.50 to 4.86) was wide, with the upper-to-lower bound ratio greater than 3.
m. Downgraded by 1 level for imprecision: The 95% CI crosses no effect (HR = 1), and the 95% CI is not narrow (>2).

A

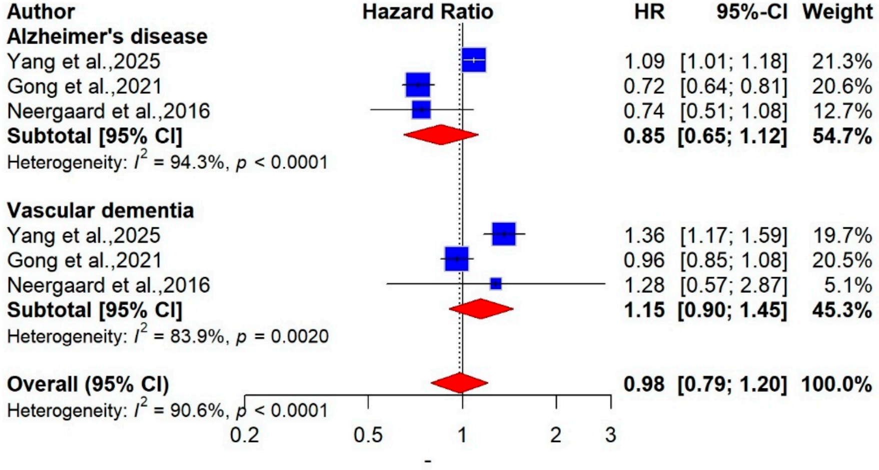

B

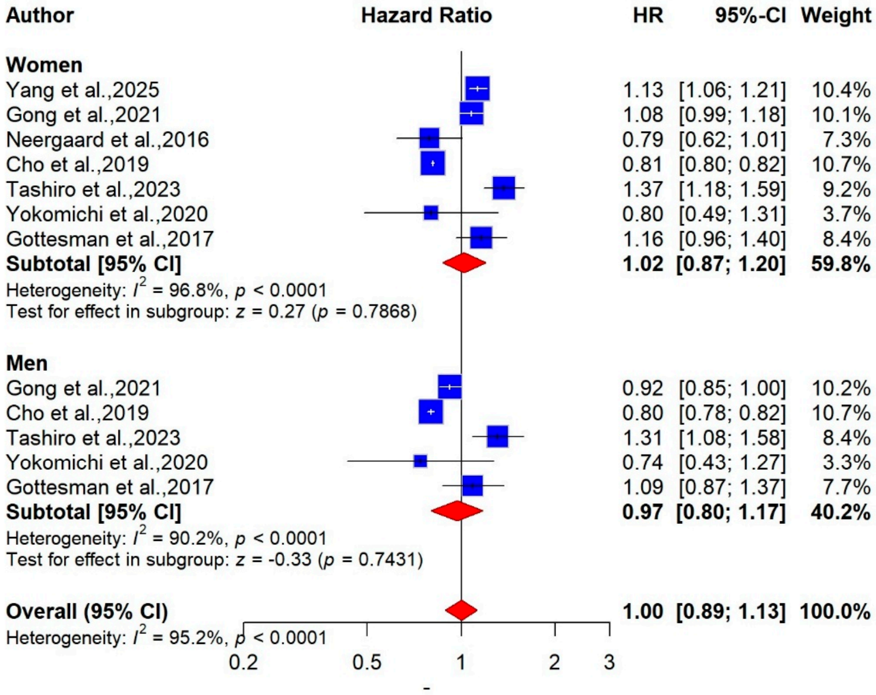

Figure S1 **A**: Forest plot of the association between BMI-defined obesity and risk of dementia types.

Figure S1 **B**: Forest plot of the association between BMI-defined obesity and risk of dementia, stratified by gender (Men vs Women).

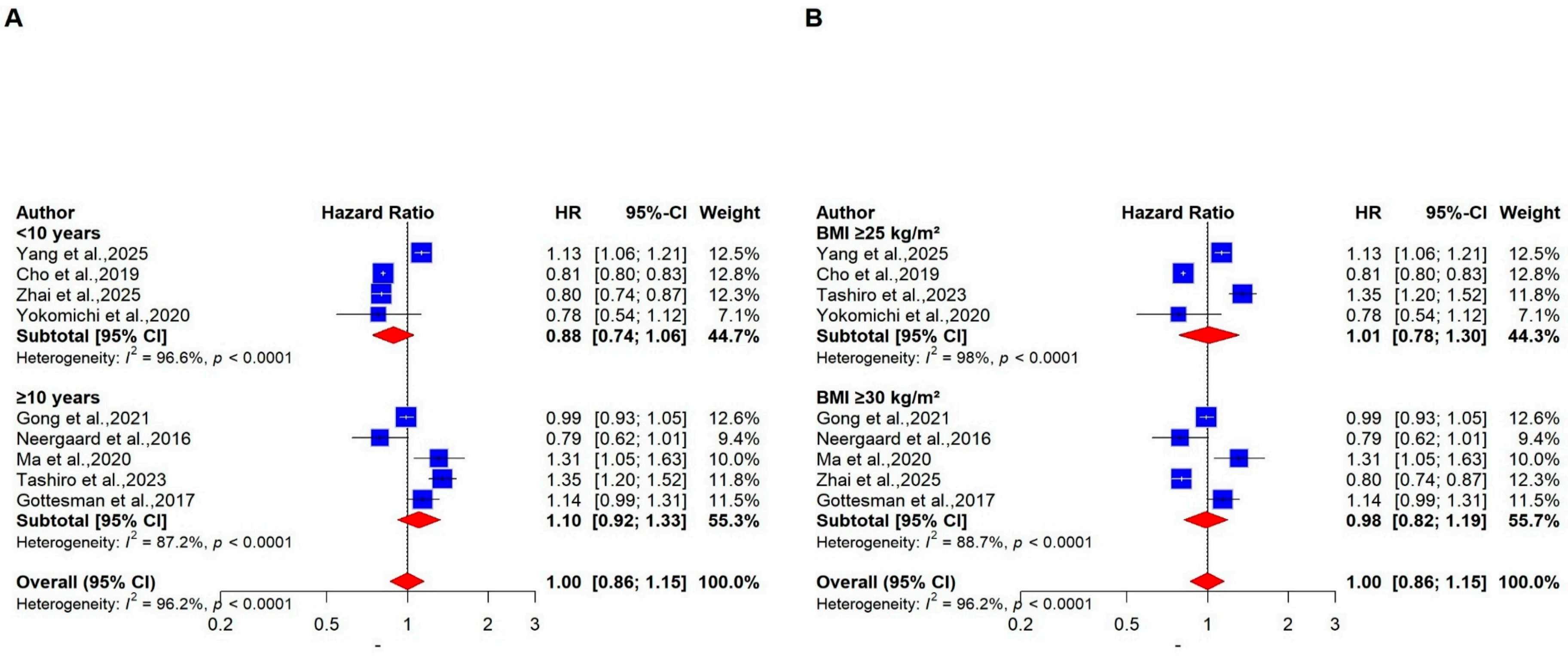

Figure S2 **A**: Forest plot of the association between BMI-defined obesity and risk of dementia, stratified by follow-up years (< 10 years vs ≥ 10 years).

Figure S2 **B**: Forest plot of the association between BMI-defined obesity and risk of dementia, stratified by BMI cut-off values (BMI ≥25 kg/m² vs BMI ≥30 kg/m²).

A

B

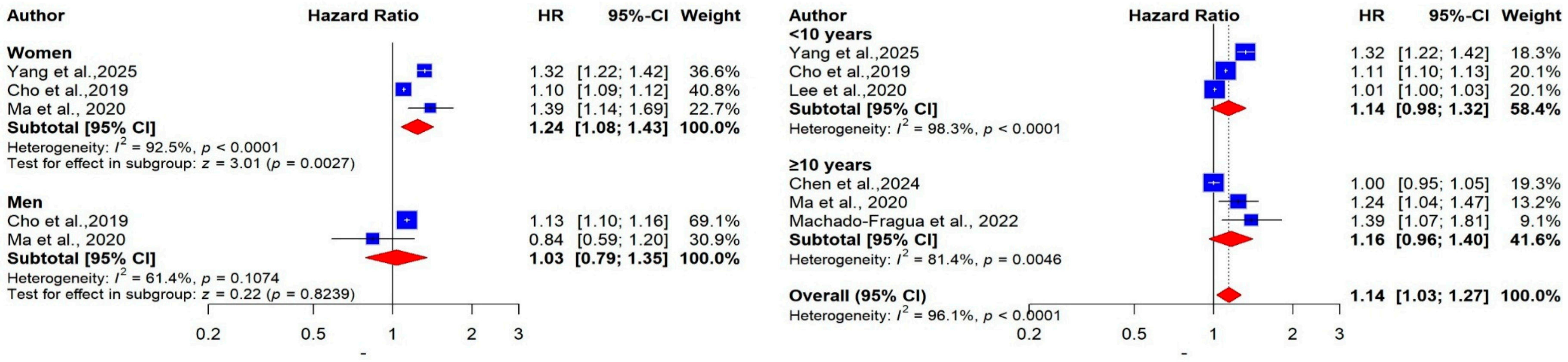

Figure S3 **A**: Forest plot of the association between WC-defined obesity and risk of dementia, stratified by gender (Men vs. Women).

Figure S3 **B**: Forest plot of the association between WC-defined obesity and risk of dementia, stratified by follow-up years (< 10 years vs $\geq$ 10 years).

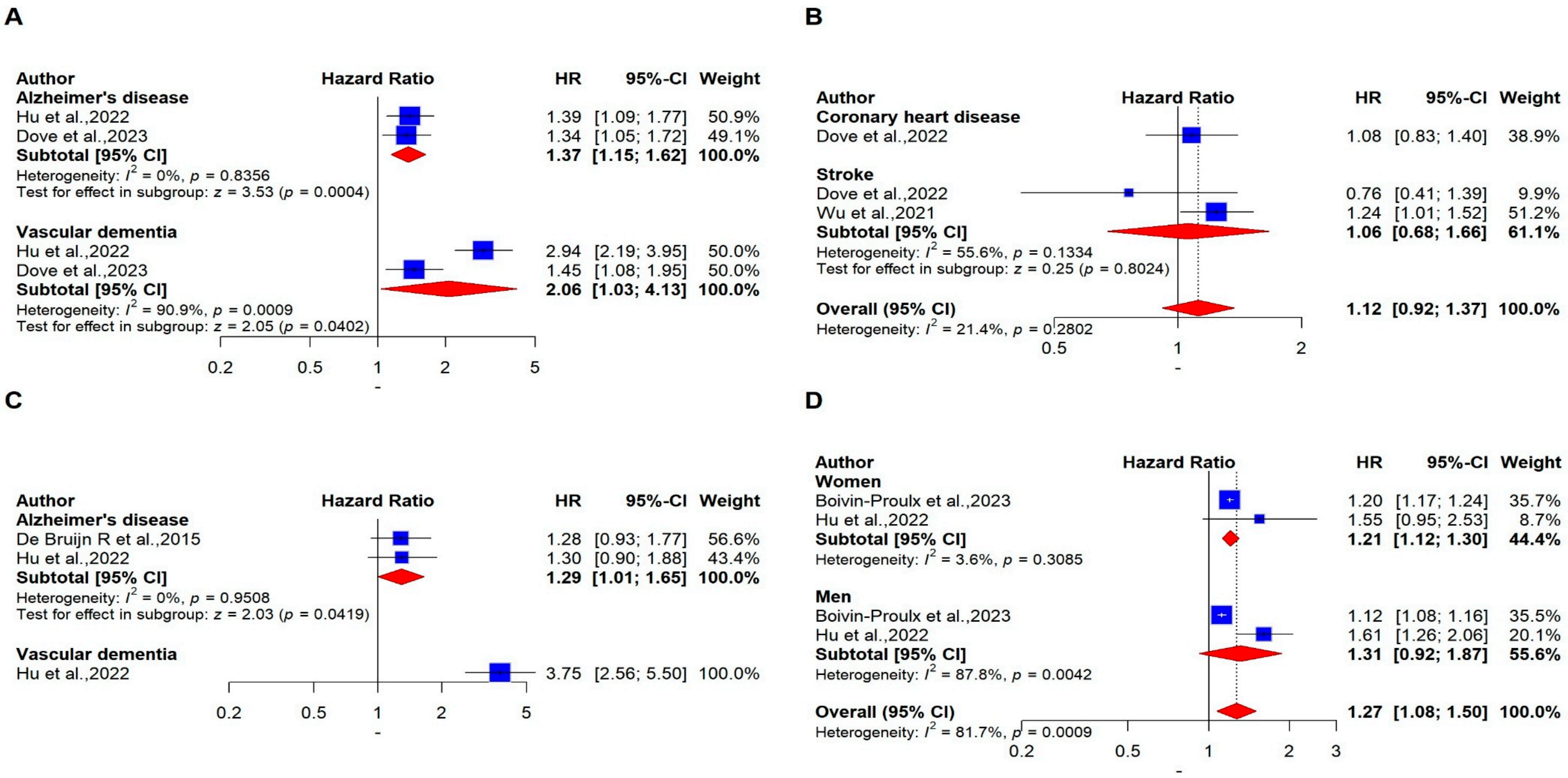

Figure S4 **A**: Forest plot of the association between coronary heart disease (CHD) and risk of dementia, stratified by dementia types.

Figure S4 **B**: Forest plot of the association between stroke and risk of cognitive dementia.

Figure S4 **C**: Forest plot of the association between atrial fibrillation (AF) and risk of dementia, stratified by dementia types.

Figure S4 **D**: Forest plot of the association between AF and risk of dementia, stratified by sex (Men vs. Women).
